# Sustainable alternative to irrigated maize monoculture in a maize-dominated cropped area: Lessons learned from a system experiment

**DOI:** 10.1016/j.heliyon.2024.e30400

**Published:** 2024-04-26

**Authors:** Christian Bockstaller, Aimé Blatz, Olivier Rapp, Rémi Koller, Sophie Slezack, Anne Schaub

**Affiliations:** a Université Lorraine, INRAE, LAE, F-68000, Colmar, France; b Association pour la Relance Agronomique en Alsace (ARAA), Schiltigheim, F-67013, Strasbourg, France; c Chambre d’Agriculture Alsace, 67500, F-Haguenau, France; d Université Lorraine, INRAE, LAE, F-54000, Nancy, France; e Chambre Régionale d’Agriculture du Grand Est (CRAGE), Schiltigheim, 67013, F-Strasbourg, France

**Keywords:** Continuous maize, Innovative cropping system, Indicators, Sustainability, Pesticide, Reduced tillage

## Abstract

Maize is the most-produced food crop in the world and is grown in intensive rotations or in monoculture (continuous maize) systems. As maize production has expanded massively across the world, many concerns have emerged about its local environmental and other global impacts. Agronomists have designed innovative cropping systems and assessed them using system experiments to make arable systems more sustainable. However, knowledge is still lacking on the sustainability of innovative cropping systems compared to highly intensive systems such as irrigated maize-based monoculture. Here, we present the assessment results of a nine-year system experiment in Alsace, France, developed to compare an innovative system based on a diversified rotation and innovative management practices (three-year rotation of maize/soybean/winter wheat (plus a cover crop) combined with reduced tillage) with a continuous maize reference system. The results cover a six-year assessment period following an initial three-year design period. Classic criteria, such as profitability, workload, pesticide use, fossil energy consumption and nitrate leaching, were assessed along with other less studied criteria, such as pesticide leaching risk, soil structure, soil chemical quality and soil biological activity. Sustainability – which includes environmental, social and economic dimensions – was assessed with the MASC 2.0 method. Overall sustainability was substantially enhanced in the innovative system (5 out of 7 sustainability classes) in comparison with the low level of the reference system (2 out of 7). This was due to a clear improvement in the environmental performance (from 2 out of 5 to 5 out of 5) while social performance was high in both systems (4 out of 5) and economic performance was low (2 out of 5) due to very low contribution to economic development. Nevertheless, the innovative system had a major drawback: lower profitability, especially when scenarios included high maize prices. Furthermore, herbicide use on maize was higher in the innovative system than in the reference one. Avenues for progress, such as encouraging stakeholder participation at the assessment stage or additional innovations such as multiple cropping, are suggested.

## Highlights

• Sustainability of alternative cropping systems (INN) to irrigated maize monoculture (REF) has to be assessed.
• A INN based on a maize/soybean/wheat rotation and reduced tillage was designed.
• A 9 years system experiment was carried out to compare sustainability of INN with REF.
• Environmental performances of INN were improved while economic ones may decline.
• Profitability for high price scenarios and herbicide use are major drawback of INN.

## Graphical abstract

**Figure:**
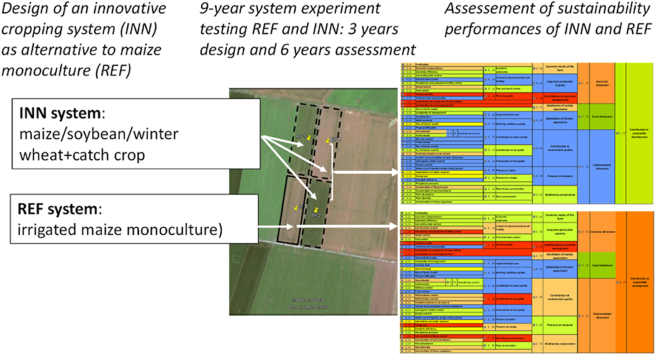

## 1 Introduction

Maize has been the leading food and feed crop produced in the world since 2004, surpassing both wheat and rice [1]. The use of modern productive hybrid cultivars resistant to diseases and synthetic inputs like fertilizers and pesticides have supported the drastic expansion of continuous maize monoculture. Although many crops see overall yield declines in short rotations [2], this has not been observed for maize in the aforementioned regions [3]. However, the negative environmental impacts of maize production have been widely documented for more than 20 years for grain maize in Europe and USA, as well as silage maize for biogas in Northern Europe ([4]; [[5], [6], [7]]) and include soil erosion, nutrient losses through leaching or runoff, pesticide transfer to water, greenhouse gas emissions, biodiversity impoverishment and more.

This increasing concern for the environmental footprint of intensive agriculture, and especially continuous maize monoculture, has led agronomists to work on designing innovative cropping systems that can meet society's demand for more sustainable cropping systems [8]. Innovation entails introducing a crop that is new for an area or a new crop management technique as well as combining existing crops and management techniques in a novel way [9]. This design step may be carried out by researchers alone, or as part of a design workshop involving different stakeholders, researchers and farmers [9] or with help of a computer [10,11]. Generally, one or more candidate prototypes are produced to be tested in a system experiment [12,13]. Using this experimental approach, the performances of the newly designed innovative cropping systems, which are described through fixed decision rules or an iterative assessment and redesign process, can be assessed and analyzed ([14]; [12]).

Alternative systems to maize monoculture have been tested in plethora of experiments, but many involve maize in rotation (e.g. Ref. [8]). No maize monoculture was studied in the review of Sandén et al. [15] who reviewed 27 long-term experiments testing alternative practices like rotation, no-tillage and compost use. Only one study out of 8 included a reference system based on non-irrigated maize monoculture in the meta-analysis of Hossard et al. [16] who analyzed the yield gap of organic systems versus integrated or conventional systems. Similarly, Lechenet et al. [12] reviewed 21 system experiments, 12 of which were based on rotations with maize, but only 1 had a treatment with continuous maize monoculture with a specific focus on herbicide resistance. Comparisons of continuous maize monoculture with rotated maize, showed in all studies a yield increase in maize in rotation vs. continuous maize monoculture [[17], [18], [19], [20]] in temperate as well as in tropical conditions [[21], [22], [23]]. This advantage of rotated maize vs. continuous maize monoculture was also observed for other agronomic themes like weed reduction [21], sensitivity to pests [22], improvement of soil properties [18,21], and environmental ones, like for instance carbon storage [24] or reduction of nitrous oxide emission [25].

However most of those studies were delivered from factorial experiments testing one factor (e.g. rotation) or two (e.g. rotation and tillage), and focused on one or two sustainability themes like productivity or soil fertility, or a specific environmental theme. An exception is the study of Alletto et al. [17] consisting of 3 on-farm comparisons of a continuous maize monoculture with an innovative rotation, assessed by a set of 15 sustainability indicators. Most indicators were improved for rotated maize vs. continuous maize except food energy production, weed pressure, Treatment Frequency Index (TFI) assessing pesticide intensity use and a calculated organic matter indicator. Likewise. Vasileiadis et al. [26] presented the results of a qualitative multicriteria assessment from a system experiments conducted over four years, comparing a conventional continuous maize with a maize/soybean rotation managed according to Integrated Pest Management strategies. They showed a trade-off between economic and environmental performances and concluded that all 3 systems were unsustainable. Finally, the study that comes closest to the scope of this article was a system experiment in Lamothe, in southwestern France [27]. The experimental layout comprised four systems tested over 3 years [27] and then 8 years [28], with irrigated maize monoculture for the reference system and a low-input maize monoculture system, a maize monoculture system in conservation tillage (other systems in conventional tillage) and a system with a three-year rotation. Like Alletto et al. [17], they performed a broad multicriteria assessment based on several measured indicators, yield, weed biomass and abundance, nitrogen supply and nutrition, herbicide use, cover crop production, gross margin, labor time, pesticide leaching, energy consumption and greenhouse gas emissions. Here again, a trade-off between technical-economic (yield, weed pressure) and environmental performances (pesticide use and leaching) was observed. However, the study focused only on maize performances and did not provided results at the rotation level. Thus, this short literature review reveals a knowledge gap regarding the assessment of the sustainability of innovative cropping systems as alternative to systems based on irrigated continuous maize rotations. Indeed, in many innovative alternative systems, maize is grown in rotation Therefore, sustainability, i.e. economic, social and environmental performances should be assessed at cropping system level, and not only at the maize crop level like in previous studies.

Here, we address the question of sustainability assessment of maize-based cropping systems by posing the hypothesis that it is possible to design innovative cropping systems that can meet a range of sustainability goals. We present the assessment results of a nine-year system experiment in the Rhine plain in Alsace, France, with a reference system based on a continuous maize rotation and an innovative system based on a diversified rotation (maize/soybean/winter wheat) and innovative management practices that include reduced tillage even in maize. Compared to most previous studies, we aimed to perform a broad assessment that addressed the main aspects of sustainability, which includes environmental, social and economic dimensions. We combined field measurements, calculated predictive indicators to perform i) an assessment of the degree of achievement of the different objectives set by the experiment committee that were used to guide the system design, ii) an agronomic assessment to analyze the degree of achievement of the objectives, iii) a multicriteria assessment of the sustainability of the systems.

## 2 Materials and methods

### 2.1 Site description

The experiment started in autumn 2009 at the Rouffach Agricultural School farm, located in the Alsace region in eastern France (47°57′43.2″N 7°19′51.6″E). More information on the socioeconomic environment of the supply chain for main crops addressed in this article and the biotic pressure in their cropped area is given in Supplementary Material 1.

The soil is classified as HYDROMORPHIC FLUVISOL according to the French soil classification [29] due to deposit from the Ill river rising in the Jura mountain range. The coarse material content is low in the arable layer but can be up to 50 % at a depth of 55–80 cm depending on the location. The substratum is an alluvial pebbly layer at around 150 cm. The average soil texture in the arable layer (0–30 cm) is 24 % clay (<0.002 mm), 40 % silt (0.002–0.05 mm), 34 % sand (0.05–2 mm) and 1.4 % coarse elements (>2 mm). The pH measured in the water is 7.4 and the organic matter is 1.8 %. Additional details are given in Table S1.

The study region has a semi-continental climate that is due to the Vosges mountains that create a barrier effect from oceanic influences. Winters are cold and dry (the average temperature in January was 0.8 °C during the experiment), while springs are wet (intensive stormy rain) and summers are warm (the average temperature in July was 19 °C during the experiment). Average annual rainfall for the year is 630 mm while evapotranspiration is 880 mm. These climatic features lead to various agronomic constraints such as irrigation, which is supported by substantial groundwater resources. Hot temperatures in June (three consecutive days with a maximum temperature of 27 °C) can be detrimental to the grain filling of wheat.

### 2.2 Design of the cropping systems

The reference and innovative cropping systems were designed according to the methodological framework set up by Reau et al. [9,30] inspired by the pioneering work of Vereijken [31] on cropping system prototyping. The framework consists of a design workshop that brings together researchers, extension engineers and farmers, and is implemented in several steps: i) a set of objectives are assigned to the innovative cropping systems to be designed, ii) the reference system is determined from an initial diagnosis and expertise on current practices in the region, iii) knowledge is collected on the cause-effect relations related to the specified objectives, the various solutions and the innovative practices to implement to meet the objectives, iv) several candidate cropping systems are developed *in silico* by establishing decision rules and determining their related practices, v) the prototypes are assessed using an *ex ante* assessment method [32] to select the best cropping system in terms of the achievement of the objectives for the experimental phase.

These steps were all implemented for our experiment. The initial steering group comprised one researcher and one technician specialized in cropping system from a research institute, one engineer from a technical institute, three engineers and one technician from an agronomic association, four engineers from the Alsatian Chamber of Agriculture, two engineers from a cooperative, five farmers belonging to the same cooperative and two agricultural school farm managers. This range of skills and expertise is advantageous: local experts understand the current context of cropping systems, while the other group members have more expertise on innovative cropping systems.

The design group identified and quantified a set of objectives covering the three dimensions of sustainability.

- One objective for the **economic** dimension: maintain or enhance profitability.
- One objective for **social** dimension: ensure a workload that allows for enough free time during the winter period for other activities (e.g. vacation, agricultural social events), i.e. 8 weeks.
- Four objectives for the **environmental** dimension: i) improve soil fertility (mainly physical and chemical properties), ii) reduce pesticide use with a Treatment Frequency Index (TFI) < 1.5 (see section 2.5.3), iii) mitigate impacts on water quality: a reduction of 30 % of nitrate leaching indicator, and for pesticides a 1 point increase of a pesticide indicator scored between 0 (negative) and 10 (positive) points scale) based on the concerns of local stakeholders and policymakers, and iv) reduce fossil energy consumption by 20 %, which is related to greenhouse gas emissions [33]. These emissions were not as acute at the start of the experiment as they are today. This last objective addresses a global impact while the first three deal with local impacts. All these references are founded on target values which were decided by the design group and which do not rest on thresholds scientifically founded [34].

### 2.3 Cropping systems tested

The reference (REF) system was a ploughed and irrigated maize monoculture without catch crops, as commonly practiced by farmers in the region at the start of the experiment. The fertilizer and irrigation strategy aimed to cover the crop's water and nutrient requirements to avoid any stress, while the crop protection strategy was implemented as insurance against any risk of pest development. The main goal for this cropping system was to maximize its gross margin.

**Fig. 1:**
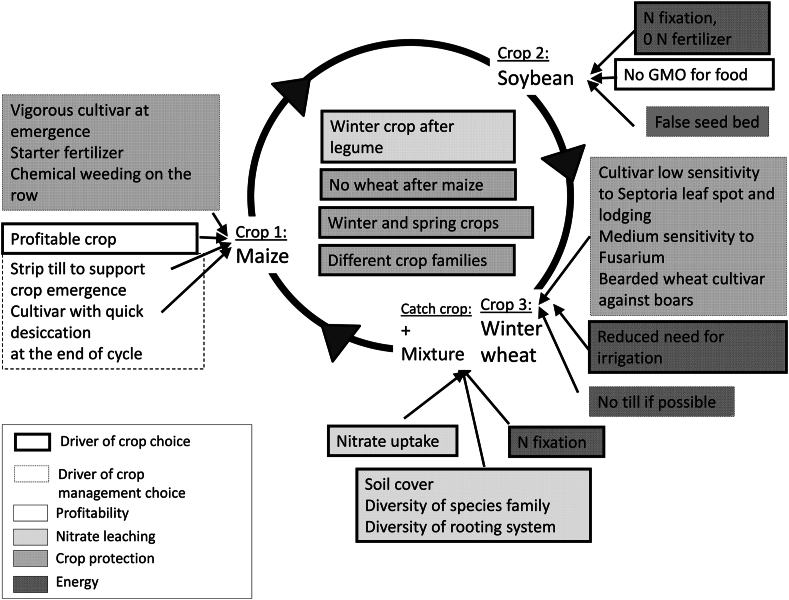
Overview of the innovative cropping system. False seed bed consists in preparing soil by one or more tillage in order to favor weed emergence to eliminate them at a young stage by another tillage.

The innovative (INN) cropping system proposed by the design workshop consisted of a three-year rotation of maize/soybean/winter wheat plus a catch crop combined with reduced tillage and reduced use of chemical inputs, especially pesticides, which the diversified rotation was expected to facilitate. Due to budgetary constraints, only one innovative system was selected. The choice of crops was driven by the availability of technical data on these crops, well known in the region to maximize the probability of success. Nevertheless, the rotation combined with systematic reduction of tillage was considered by participants of the design workshop as very original for the region. Furthermore, the catch crop consisted in a mixture of species with a high proportion of legumes (e.g. 17 kg spring vetch, *Vicia sativa* 2.5 kg phacelia,Phacelia *tanacetifolia*, 3 kg sunflower, *Helianthus annuus*), while in many fields only mustard catch crop can be observed. Fig. 1 provides an overview of the innovative system with the underlying decision-rule drivers. An example of detailed weed control strategies can be found in Fig. S2. Similar figures (not shown) were designed for disease and lodging, pest control and nitrogen supply to crops.

The experiment was launched in 2009 for an initial year to test the cropping systems. During first complete rotation (2010–2012), the design group adapted the decision rules and stabilized the cropping system design.” Please provide some examples of adaptations implemented during this period. One example of adapting the decision rules involved abandoning grass undersowing in maize because of failures in the development of this catch crop (lack of light, pests, etc.).

### 2.4 Experimental design

The system experiment was conducted on four fields with an average size of 0.61 ± 0.04 ha: one REF field and three INN fields for the three crops in the rotation (Fig. S1). Each rotation term was cropped each year so that the experimental layout consisted of temporal replication according to the typology in Lechenet et al. [12]. As usually practiced in system experiments, the fields were large enough (between 0.56 and 0.66 ha) to be able to use farm equipment.

### 2.5 Evaluation of the economic, social and environmental objectives

#### 2.5.1 Profitability

Profitability was assessed by the calculation of a semi-net margin using the CRITER software [35]. The semi-net margin was calculated for eight contrasting real-price scenarios as in Lechenet et al. [36] to assess the effect of price volatility. The price scenarios integrated harvested crop and fertilizer prices and were based on real data between 2007 and 2014 (Table S3). <

#### 2.5.2 Workload

Workload was calculated by assessing the total duration of each field operation by means of the CRITER 5.4 software [35]. Detailed software outputs make it possible to calculate the total workload (hours/ha/year) while the distribution throughout the year was assessed in an Excel file to identify workload peaks.

#### 2.5.3 Reduction of environmental impacts

Soil fertility was assessed by monitoring chemical soil properties at the start (2009) and end (2018) of the experiment. Soil organic matter and main nutrient contents (P, K) measured on soil samples from the tilled layer at depths of 0–10 cm and 10–25 cm according to standard laboratory protocol. Physical fertility through soil structure was assessed visually by the method described in Roger-Estrade et al. [37] (Supplementary Material 4). Soil microbial enzyme activities and extractable soil C and N were assessed according to Ref. [38] (Supplementary Material 9).

Pesticide reduction is addressed by the treatment frequency index (TFI) [39], currently used in the French national pesticide reduction plan (Ecophyto) and is calculated (see Supplementary Material 3). Pesticide transfer to groundwater, surface water and air was assessed by I-Phy3 (v1.70) from the INDIGO method (Pierlot et al., 2023, see Supplementary Material 5). For nitrate leaching, we used the nitrogen indicator (I–N v.2.70) from the INDIGO method ([40], see Supplementary Material 6). Energy consumption was calculated using the I-En indicator from the INDIGO method [41].

The semi-net margin and TFI were calculated using the CRITER 5.4 software [35], while I-Phy, I–NO_3_, I and I-En indicators were calculated using specific Excel sheet calculators to benefit from the latest version of the calculation method (not available in CRITER 5.4.).

### 2.6 Agronomic evaluation

The agronomic evaluation aims to complete the evaluation of the objectives and provide some explanatory details. Yield is the major factor of the semi-net margin and determines the production term while grain quality assessment in terms of mycotoxin contamination may influence the price (this factor was not considered here). Yield was measured by weighting the amount of grain harvested by the combine harvester on each field and normalized for each crop at 15 % grain humidity. Yield components (number of plants, number of ears, thousand kernel weight) were also measured for maize throughout crop development. Mycotoxin contamination was assessed on sample in accordance with best practices and analyzed in a certified laboratory (Capinov, located in Landerneau, France). Weed growth was assessed visually before harvest and species showing a high density were recorded. Several pests and diseases were monitored visually from seed to harvest on plots within each field. Nitrogen content was measured in maize grains and plants, and water supplies were assessed by monitoring soil water tension using tensiometers. Nitrogen uptake by the cover crop planted after the wheat harvest was measured in plant samples. See Table S7 for more details.

### 2.7 Multicriteria assessment of sustainability

Finally, we performed a multicriteria assessment of sustainability by means of the MASC 2.0 method [42] supported by the DEXi software [43]. Through this formalism, MASC conceptualizes the sustainability assessment problem by breaking it down into the three typical dimensions used to define sustainability: social, economic and environmental. For each dimension, indicators are organized into a tree-like structure (decision tree) created using 38 basic indicators and 26 aggregated indicators. All the indicators are expressed in qualitative classes (e.g., low, medium, high) after discretization of the quantitative indicators. Results are expressed as a synoptic table representing the decision tree, from the basic indicators through the different aggregation levels and finally to the global sustainability indicator (see Fig. 5a and b). Details on the basic indicators are given in Table S8.

### 2.8 Statistical analysis

Due to the lack of spatial replication, we did not carry out a statistical test like an ANOVA, but provided only descriptive statistics, calculated with Microsoft Excel Sheet. Temporal replications made possible an analysis in terms of the frequency with which the REF system was exceeded by the INN system. For the assessment of soil biology quality, a principal component analysis was performed with R software [44], (see Supplementary Material 9).

## 3 Results

### 3.1 Global evaluation of objective achievement

**Fig. 2:**
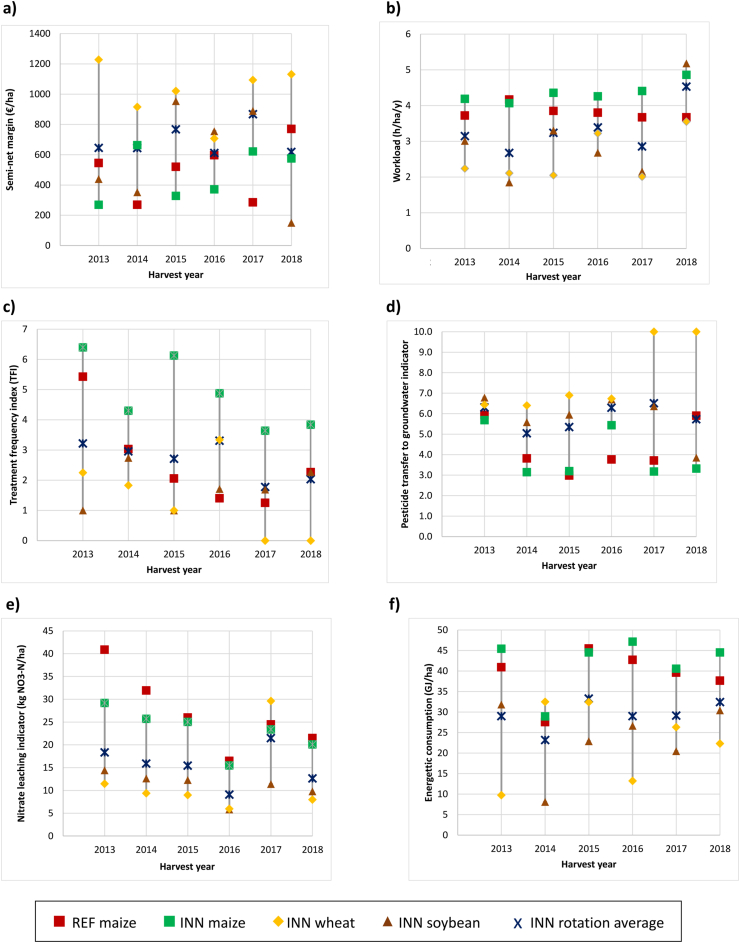
Main sustainability performance indicators of the reference (REF) and innovative (INN) cropping systems with detailed results by crop for the innovative system: (a) semi-net margin (euros/ha) for profitability, (b) workload (h/ha/y) for quality of life, (c) treatment frequency index for pesticide use, (d) pesticide transfer to groundwater indicator (0 = high risk, 10 = low risk) for water quality (pesticide), (e) nitrate leaching indicator (kg N–NO_3_/ha) and f) energy consumption indicator (GJ/ha) for energy use. For (a) and (d), the higher the result, the better the assessment. For (b), (c), (e) and (f), the higher the result, the worse the assessment. The bars represent the range of variation of the INN system within a year for a given indicator. Results were analyzed in terms of the frequency with which the REF system was exceeded by the INN system.

Fig. 2 shows that the innovative (INN) cropping system performed better than the reference (REF) system on average with regard to the main sustainability indicators assessing the achievement of the objectives set for the INN system. The INN system outperformed the REF system every year for the nitrate leaching and energy consumption indicators, 5 out of 6 years for the workload indicator, and 4 out of 6 years for the pesticide transfer to groundwater indicator. For the treatment frequency indicator (in maize mainly treated with herbicides), the REF system outperformed the INN system for 2 years of the study, both systems yielded similar results for 3 years, and the INN system did better than the REF system for 1 year (the first). For the semi-net margin, the REF and INN produced similar results in 3 years of the study, and the INN performed better for 2 years; the average semi-net margins for the six-year period were thus very similar for both the REF and INN systems (scenario 1 on Fig. S3). However, comparisons between the maize crop in both systems did not always favor the INN system. In several cases, the wheat crop explained the better INN system results.

**Fig. 3:**
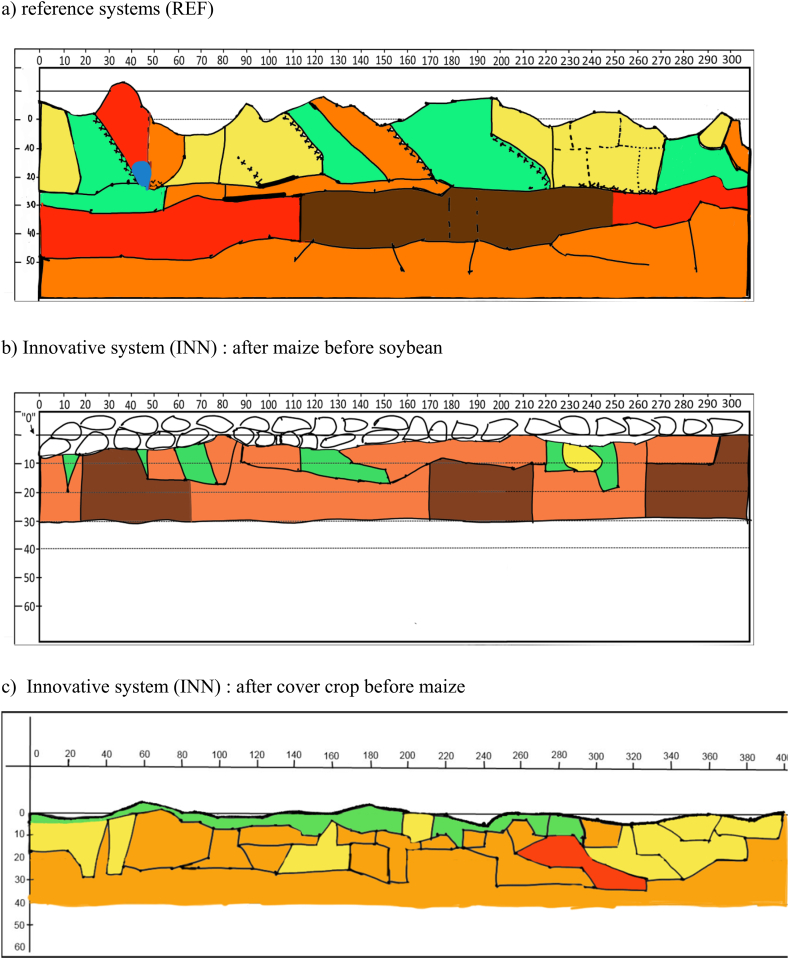
Description of soil structure for the reference and innovative systems. The method is detailed in Supplementary Material 4 and the colour code in Table S4. Green: (very) favorable, yellow: rather favorable, pale orange: slightly favorable, dark orange or red: unfavorable, brown: very unfavorable, blue: very unfavorable (hydromorphy). (For interpretation of the references to colour in this figure legend, the reader is referred to the Web version of this article.)

Overall, the INN system performed better than the REF system in terms of soil fertility components such as soil structure (physical component) and soil organic carbon (chemical). For biological activity, no difference was observed between maize in the REF and INN systems, but components such as protease activity and the size of the biological compartment tended to be higher for wheat and soybean in the system (Table 1). For other chemical components such as pH (in water) and P and K nutrients, the trend was not as clear. In the absolute assessment, the results were not as favorable. Regarding soil structure, there were still some areas with a very unfavorable structure in the INN system in maize after soybean, while the situation improved in the field after wheat but without many very favorable or favorable areas (Fig. 3a, b, 3c). This is confirmed by the soil organic carbon results. When the impact on soil structure quality is considered, the ratio with clay is a better indicator of soil structure quality than the absolute value of soil organic carbon [45]. Despite improvement in the INN system, the ratio remains at an “unfavorable” level in all cases (Table S1).

**Table 1:**
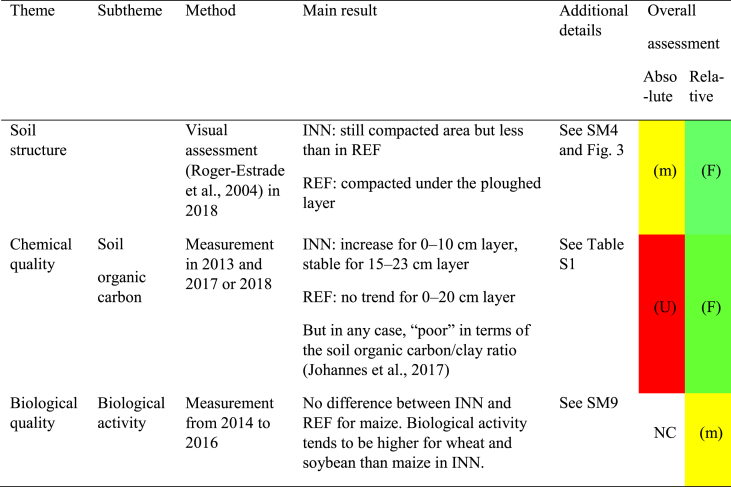
Assessment of impact on soil fertility components for the reference (REF) and innovative (INN) systems. The absolute assessment provides the absolute sustainability performance of the INN system: (F) or green shows a favorable result, (m) or yellow: a medium value or the lack of a clear trend, (U) or red an unfavorable value; The relative assessment compares sustainability of the INN system with the REF system: (F) or green means that INN system performs better than REF, (m) or yellow that both systems are similar, (U) or red that REF system performs better than INN, NC: not concerned.

**Fig. 4:**
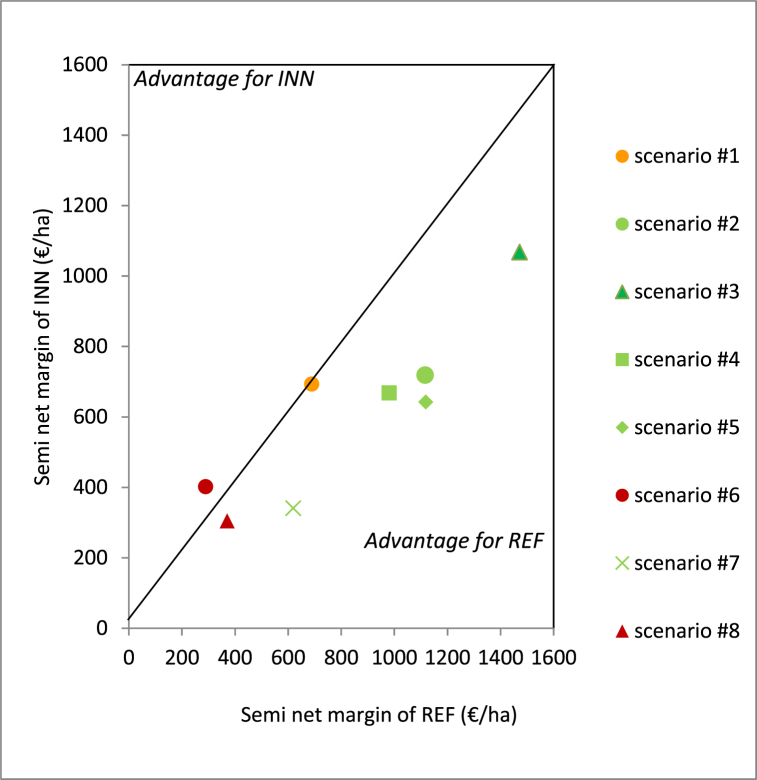
Average semi-net margin (SMN) of the two systems, reference (REF) and innovative (INN) in function of price scenarios (Table S8).

An in-depth analysis of the results by indicator shows that the difference between the REF and INN systems varies considerably for the semi-net margin for the eight price scenarios for outputs and inputs (Fig. 4). The semi-net margin logically increases as the maize grain price increases. For unfavorable price scenarios or average prices – as in scenario #1 used in Fig. 2a – both systems either performed the same or the INN system performed better (scenario #6), except in scenario #7. This last scenario, which had the lowest soybean price, strongly impacted the semi-net margin; in 2014, this was due to low yields (Table 2), while in 2013, 2016, and 2018, high irrigation costs for the soybean crop were not offset by the final product. For the four favorable price scenarios for maize, the REF system systematically performed better. Overall, the REF system performed better than the INN system in five out of eight scenarios. Across the eight scenarios, variation of the product (between −€561 and +€39/ha in favor of INN) was much more variable than total costs (between −€86 and −€54/ha, always in favor of INN), which highlights the role of maize prices. Finally, the semi-net margin variation was slightly lower in the INN system (variation coefficient 42 %) compared to the REF system (variation coefficient 49 %).

**Fig. 5:**
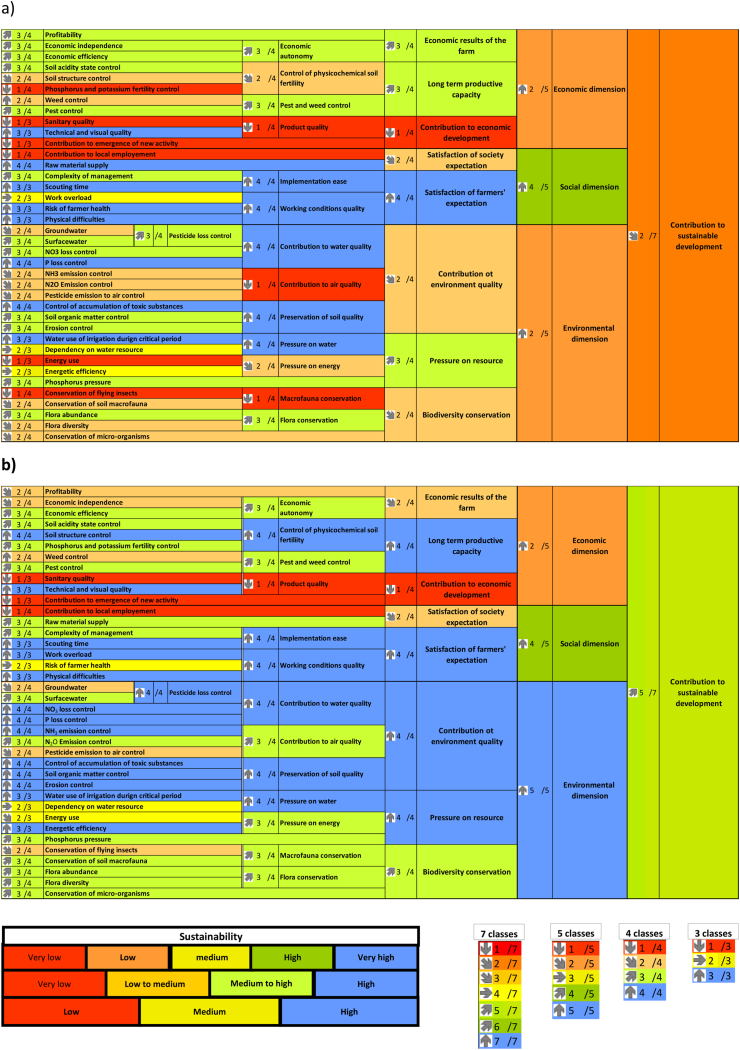
Synoptic presentation of the results from a multicriteria sustainability assessment with the MASC 2.0 model; (a) depicts the reference system and b) the innovative system.

**Table 2:** Yield (T/ha) of the crops for the two systems, reference (REF) and innovative (INN).

	2013	2014	2015	2016	2017	2018	Yield target
REF							
Maize	10.33	9.82	12.60	10.00	12.17	11.92	11.00
INN							
Maize	10.75	10.85	11.20	10.70	12.14	12.31	11.00
Soybean	2.97	1.54	3.73	3.32	3.21	2.89	3.00
Winter wheat	6.92	7.77	8.0	4.68	7.37	7.43	7.00

For workload, the INN system performed better than the REF system on average, except in the last year (Fig. 2b). This result is explained by the lower workload for soybean and wheat in the INN rotation compared to maize in the REF system; meanwhile, the maize workload in the INN system is nearly always higher than for maize in the REF system. This was mainly due to the additional workload needed to sow and destroy the catch crop after wheat harvest, which was considered as belonging to the maize cropping period. One exception was in 2018, where the REF system has a lower workload than the INN system. This was explained by the need to resow the soybean crop after ravens caused considerable damage before emergence. It should be noted that time spent for irrigation was not included in this calculation. If this workload had been included, the difference would lean more in favor of the INN system because winter wheat requires less irrigation than maize.

With regard to pesticide use, the average trend was not as clear as for the workload indicator (Fig. 2c). Aside from the first year in 2013, the REF system performed better on average than the INN system in 2015, 2016 and 2017, and the two systems were similar in 2014 and 2018. However, maize in the INN system was always the most-sprayed crop and had the highest TFI, while wheat was the least sprayed. This was especially true in 2017 and 2018, which saw dry springs and low weed pressure, meaning that wheat did not have to be sprayed.

The risk of pesticide transfer to groundwater was higher for the REF system on average than for the INN system for 4 years and at the same level in 2014 and 2018 (Fig. 2d). Maize in both the INN and REF systems performed more poorly than the other crops for 4 years, while in 2013 all results were similar and in 2018 the soybean result was exceptionally low. The high transfer risk, expressed as an indicator value between 3 and 4 for maize in the REF and INN systems as well as for soybean in 2018, can be explained by the use of the active ingredient S-metolachlor, while the value of around 6 in maize was due to use of the active ingredient nicolsulfuron (Table S10). In soybean, the value of around 6 can be explained by the use of the active ingredient imazamox; the value was lower when the active ingredient was applied earlier in May, since the soil is potentially wetter than in June, which favored pesticide transfer to groundwater. In wheat, the indicator value of nearly 7 was due to the use of fluroxypyr (in 2013 and 2014), prochloraz (2013 and 2016), iodosulfuron-methyl-sodium (in 2015), and 2,4-D (in 2016). The application of glyphosate on maize in the INN system or on soybean yielded an indicator value of around 6 (minimum 5.7 and maximum 8.0) depending on the application rate. This best (maximum) value for glyphosate was due to low application rate and later application date.

Nitrate leaching was lower in the INN system than in the REF system, although in 2016 the values were low and quite similar for all crops (Fig. 2e). The amount of drainage explained the variation between years. Dry winter conditions in 2016–2017 and 2018–2019 with drainage of 40 mm and 71 mm, respectively, explained the low leaching in general for all crops for these years. Differences between systems were due to lower nitrate leaching after soybean and wheat than after maize. For maize in the INN and REF systems, the indicator did not yield any difference; this is because in both systems, fertilizer use did not exceed the crop requirements and the fallow periods after maize were managed in the same way. The lower leaching after wheat and soybean can be explained by the catch crop sown after wheat and before maize and the soybean-wheat sequence that had lower mineralization after soybean and a slight uptake before winter by the following wheat crop. One exception is the high leaching after wheat in 2017, which reached the same level as maize. This was due the poor growth of the catch crop, which was sown later and produced a low yield (less than 0.5 t DM/ha).

For fossil energy use, the better performance of the INN system compared to the REF system (Fig. 2f) was explained mainly by its lower energy consumption, which was mostly due to a no nitrogen mineral fertilizer required for soybean. Fossil fuel used to make mineral fertilizer amounted to 68 % and 59 % of the total use for the REF and INN systems, respectively, with the INN system showing a 43 % reduction in energy use compared to the REF system. This reduction met the mineral N fertilizer reduction objective of 40 %. This major contribution of N fertilizer to energy consumption has been well documented for some time [41]. The second main contributor is energy consumed for equipment use in fields, which accounts for 33 % and 27 % of the total use for the REF and INN systems, respectively. This smaller reduction means that total energy was reduced by only 35 %, compared to the 40 % reduction for N fertilization. Between crops, energy use in maize was the highest in both systems, with a slightly lower consumption in the INN system for 3 out of 6 years. Wheat showed an intermediate and variable performance.

Table 3 shows that five of six objectives were achieved by the INN system, including the social objective and the four environmental objectives. For the economic objective assessed by the semi-net margin, both systems perform similarly when an average price scenario is considered. Reduced pesticide use was the only failure with regard to the environmental objectives due to the maize crop (Fig. 2c). The REF system achieved the objective for two years (2016 and 2017). The TFI and workload were the only objectives expressed according an absolute value based on a reference value for maize. The other objectives were expressed as relative values compared to the REF system, for water quality (pesticide and nitrate) with a quantification and the three other objectives with a vague statement, doing better than the REF system. For soil fertility, the objective was achieved although the absolute assessment did not yield acceptable values in terms of sustainability. Finally, the catch crop growth after wheat yielded an acceptable performance (aboveground biomass >1.5 T dry matter/ha) in 5 out of 6 years, except in 2018 when the cover crop was sown too late in November after dry conditions in late summer and early autumn.

**Table 3:**
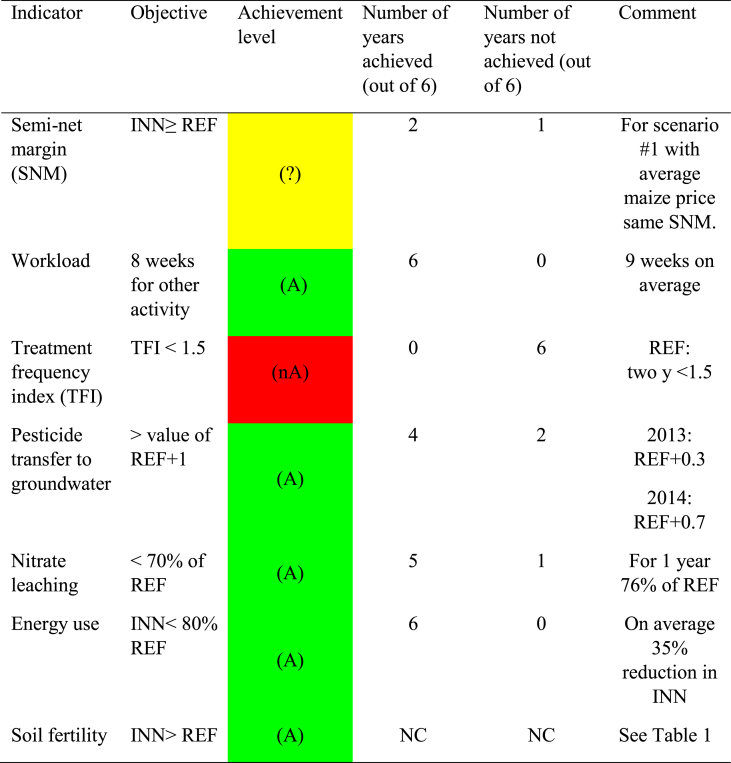
Evaluation of objective achievement for the innovative cropping system. (A) or Green: achieved, (?) or yellow: partially or downward trend, (nA) or red: not achieved. INN = innovative cropping system, REF = reference cropping system, NC: not concerned.

### 3.2 Agronomic evaluation

For maize yield, the INN system outperformed the REF system 4 years out of 6 and performed similar to the REF system 1 year out of 6 (Table 2, Table 4). The INN and REF systems reached the yield objective 3 years out of 6. Winter wheat yield was poor in 2016 due to very unfavorable wet climatic conditions in May and soybean yield was affected in 2014 because of weeding failure and hare attacks.

**Table 4:**
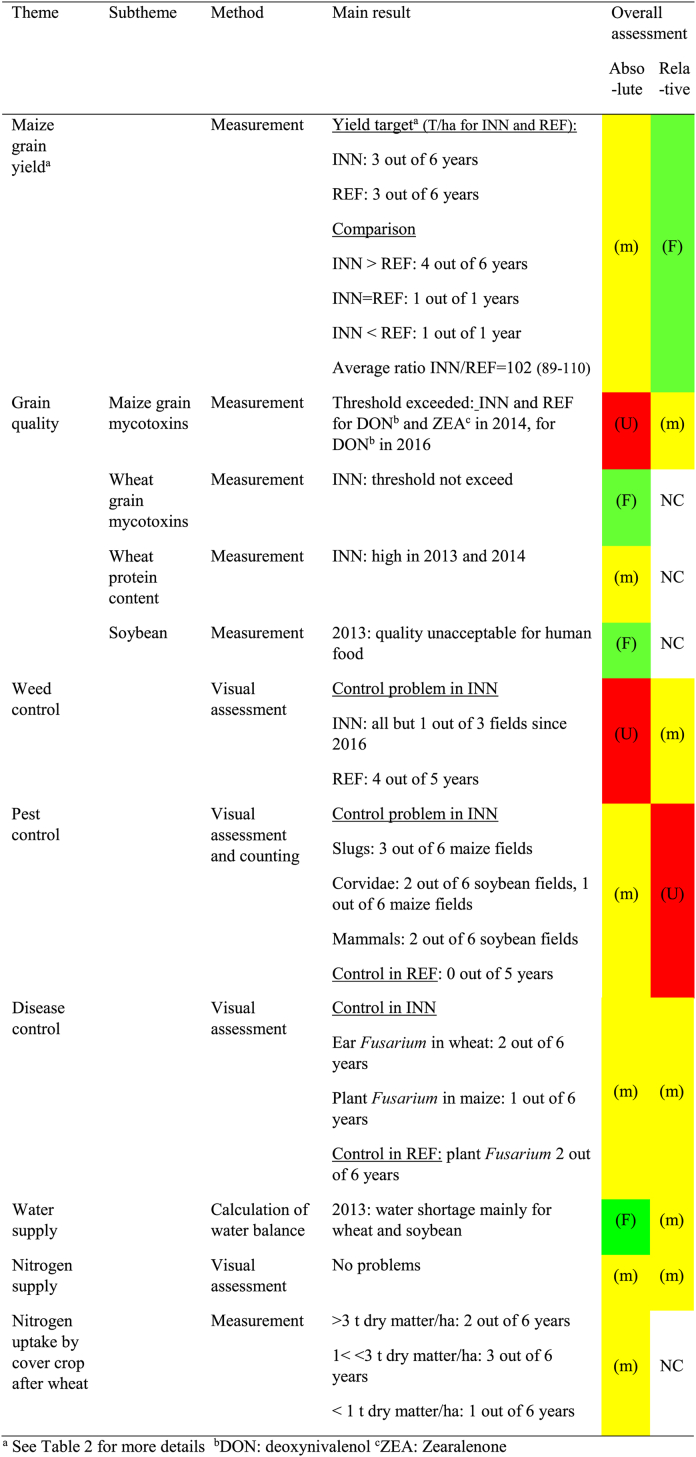
Assessment of agronomic components for the reference (REF) and innovative (INN) systems. For the absolute assessment, (F) or green indicates a favorable value, (m) or yellow indicates a medium value and (U) or red an unfavorable value; for the relative assessment, green means that the INN system performed better than the REF, yellow that both systems are similar and red that the REF system performs better than the INN system, NC not concerned.

For harvest quality, both systems yielded similar results regarding mycotoxins in maize, which occurred 2 years out of 6, the same year in both systems. For wheat, no harvest problems like mycotoxin content, specific grain weight occurred in the INN system, except in 2016 for the specific weight due to very wet conditions before harvest. The protein rate in wheat was high only 2 years out of 6, a result that could be improved upon. For soybean, the harvest quality was not analyzed.

While nitrogen and water supply were at an acceptable level in most years, weed control was undoubtedly the weak point in both systems, especially in the INN system. Some grass species (e.g., *Echinochloa crus-galli*, *Setaria viridis*), dicotyledons (e.g., *Chenopodium album*, *Persicaria maculosa*, *Sonchus* sp.) and perennial species (e.g., *Convolvulus arvensis*) were difficult to control.

Conversely, pest control issues occurred with slugs, ravens and mammals in the INN system but not in the REF system. Diseases were also a problem, mainly ear (wheat) and plant fusarium (maize), with the latter affecting both systems. In some years, this led to additional pesticide treatments in the INN system against slugs: 2013 and 2015 in maize (three treatments) and in 2014 in wheat and soybean. Fungicide use in wheat was managed depending on the climate-induced pressure dependent: none was required in 2015, 2017 or 2018.

Finally, with regard to nitrogen management, catch crop growth was good, with only one year showing a real problem due to a delayed sowing date. As a result, the nitrate leaching indicator after wheat showed a favorable environmental value for 5 out of 6 years.

### 3.3 Multicriteria assessment

The INN system showed better overall sustainability than the REF system, rising three classes (from class two to class five on a seven-class scale (Fig. 5a and b)). While this gain is appreciable, the remaining two classes highlight potential for further improvement. As it can be seen from Table 3, the improvements applied to the environmental dimension, whereas sustainability remained low for the economic dimension and high for the social dimension in both systems. For the social dimension, differences between both systems at a lower level of aggregation were slight. At a lower level of aggregation, an inversion occurred between the “work overload” and “risk for farmer's health” criteria. “Work overload” ranked in the “low” to “medium” class in the REF system and “high” (on the sustainability scale) in the INN system, while this was inversed for the “risk for farmer's health” criterion. The economic dimension produced a much greater difference than the social dimension, in spite of the same result at the “dimension” level (i.e., the highest aggregated level). For the economic dimension, “profitability” declined while “long-term productive capacity” was enhanced in the INN system (both with a difference of one class), which explain the same result at a higher aggregation level. The shift for the “long-term productive capacity” was explained by the improvement in “control of physicochemical soil fertility,” and more specifically at a lower level of aggregation by the “soil structure control” criterion, which was partially confirmed by the soil structure observation (Table 1 and Fig. 3a, b, c).

For the environmental dimension, improvement comes from the “contribution to environment quality,” with an increase of two classes, while the “pressure on resource” and “biodiversity conservation” criteria showed an increase of two classes. The sustainability enhancement for the “contribution to environment quality” criterion was explained by the “air quality” criterion, which stemmed from a substantial decrease in nitrogen-to-air emissions linked to a 40 % reduction in nitrogen fertilization when soybean was introduced into the INN system. For the “pressure on resource” criterion, improvement came from the “pressure on energy,” as already analyzed above (Fig. 2f). For biodiversity, the enhancement of the INN system was due to better performance for the “conservation of macrofauna,” “flora diversity” and “conservation of microorganism” criteria at a lower level of aggregation. Two drivers played a major role in these enhancements of biodiversity criteria: first, crop diversity, and second, the reduction of tillage, even though pesticide use did not decline in the INN system, nor did the TFI of herbicides or the TFI of insecticides.

## 4 Discussion

### 4.1 Performance of the innovative system

Our system experiment on maize-based rotations in high-yield irrigated maize monoculture systems delivered new knowledge on the sustainability of innovative rotations. As our literature review in the introduction shows, very few studies have focused on the improvement of such reference systems, even though they are important in some regions (like Alsace in France). Overall, the newly designed system showed improved sustainability due to significant enhancement of environmental performances and stable high social performances, while economic results declined when price variability was taken into account. Nearly all nine experiments conducted in Europe that were assessed using the DEXiPM method with ex post data from field experiments [26] produced similar performances.

When compared to the reference system, sustainability of the innovative system increased of three classes. According to the seven-level MASC scale, this reflects a jump from the second to the fifth level. While this is a substantial improvement in sustainability, the two remaining levels show there is further progress to be achieved, and the innovative system did have its weak points. First, economic sustainability did not improve and the pesticide reduction objective was not achieved. Additionally, pesticide and herbicide treatments were applied at a higher frequency in the innovative system. In terms of economic performance, no change was observed when the average price scenario was considered, unlike in the three systems studied by Davis et al. [8]; however, they did not include a maize monoculture crop. When other price scenarios are considered, the innovative system performed worse in five out of eight scenarios, while Giuliano et al. [27] found a decrease in performance of 74 % for the three-year rotation when using the actual prices of each harvest year. Profitability certainly remains a major criterion for farmers, which means they may not consider the system acceptable. The small reduction in the economic result variability (with a variation coefficient of 49 % and 42 % for the REF and INN systems, respectively) may not be a sufficiently convincing argument in favor of the innovative system.

The increase in pesticide use seen in the innovative system (see below for explanation) was observed by neither Davis et al. [8] nor Giuliano et al. [27]. In Vasileiadis et al. [26], all the experiments resulted in reduced pesticide use, with a much greater reduction in the experiment without maize. This was probably because the degree of crop rotation diversification was lower in maize than in the innovative wheat-based rotation. Getting stakeholders to accept this innovation could be a major hindrance within the scope of the French Ecophyto plan to lower pesticide use. However, considering the results of the I-Phy indicator for our experiment, an improvement in water quality due to less pesticide leaching is expected. Vasileiadis et al. [26], who implemented the latest version of the Synops indicator, also obtained a similar result for two out of the three sites (with no changed observed for the third). Giuliano et al. [28] found the same trend in measurements on the Lamothe site during an eight-year experiment. Thus, Giuliano et al. [28] measured leaching peaks for the S-metolachlor active ingredient, which the I-Phy indicator also identified as risky, as well as mesotrione, which I-Phy does not consider as a risky substance (Table S10). The discrepancy may be explained by the existence of strong preferential flows on the Lamothe site which strongly increases the leaching risk of that active ingredient [46].

Regarding other environmental performances, the innovative system outperformed the reference system in soil fertility, nitrate leaching and energy consumption. The calculated concentration ensured a result for the innovative system that was much lower than the standard of 50 mg NO_3_/L, which water managers may consider acceptable. As for energy, the 35 % reduction calculated for the innovative system was much higher than the 7 % reduction observed by Giuliano et al. [27]. If we assume a proportionality of fossil energy use with greenhouse gas emissions, the 35 % decrease is not enough, but it does come close to the target of 40 % set by the French National Low-Carbon Strategy [47].

This mixed performance must be analyzed with regard to the innovation introduced in our experiment's innovative system. In our system, a lever of diversification was associated with reduced tillage for maize (chisel or strip-till) and chisel tillage for soybean and in some cases no-till for wheat, while in the experiment of Giuliano et al. [28], both conventional and diversified monoculture systems were ploughed. Reducing tillage of maize is a challenge in high-yield areas. Nevertheless, unlike Giuliano et al. [28], and Pittelkow et al. [48], we did not observe lower yields (Table S9). The main problem was weed control, which is often the case in reduced tillage or no-till systems [49]. The difficulties with weeding may also be explained by the location, which had high weed density in maize and a high proportion of sugar beet in the rotation before the experiment. Both these crops have the highest herbicide use intensity, which may reflect their sensitivity to weeds [50]. Regardless of the underlying reason, weed pressure resulted in higher herbicide use, which increased costs and lowered profitability. Spraying less of an active ingredient with a high application rate (around 1 kg/ha/treatment) such as S-metolachlor made it possible to lower the risk of leaching, but replacing by a product like nicosulfuron did not eliminate all risk, so the indicator value did not reach the “medium to high” sustainability class.

For the other environmental performances, the improvement came mainly from replacing maize with other crops rather than from the maize itself. The major driver was the lower use of inputs, especially nitrogen in soybean and pesticides in wheat. For wheat, the given context with a high rate of spring crops, an increasingly frequent dry spring conditions and no problems with pests such as aphids resulted in a low TFI in this crop, and 2 out of 6 years did not require any treatment at all. The introduction of winter wheat facilitated the sowing of a cover crop during the fallow period, a major lever to reduce nitrate leaching [51]. This role of diversifying crops was also observed for most of the soil fertility criteria measured annually in crops. This was the case not only for the biological measurements but also for physical properties determined by visual observation of soil structure, which showed better results after wheat than after maize (Fig. S4).

Finally, the results we obtained at field level should be completed by an assessment at the regional level to include impacts on the whole agri-food chain [14]. This is important, especially when the designed innovative system would be extended on a substantial area in a region with a well-established agri-food chain centred around maize monoculture. In particular, the costs of reorganization due to the introduction of new crops and the reduction of the main crop harvest must be addressed because they are a main driver of the socioeconomic lock-in observed with diversification [52]. Such studies may involve the assessment of scenarios on upscaling the innovative system (percentage of area, location, etc.). This can be achieved through a specialized study or an integrated assessment and modeling platform like the MAELIA platform [53].

### 4.2 Methods of result acquisition

According to the typology in Lechenet et al. [12], the implemented experiment may be classified as a prospective experiment with innovation going further than slight adaptations to current systems. A rotation with three crops combined with reduced tillage is a significant change in an area where every crop sequence includes a high percentage of maize or even monoculture, given that maize fields are ploughed every year in most cases. Results may continue to be disappointing for some criteria such as profitability, which may make it difficult to get farmers to accept the change. However, the experiment may provide a dataset of results for similar systems that could be further improved and become profitable if a scenario of low price for grain maize would last for several years. Because of the systemic design, we were unable to clearly determine the effect of individual factors (e.g. weed pressure), but we have made assumptions that could be tested in other more analytical designs. After an initial design phase to allow for comparison between both systems, and when general decision rules are considered (Fig. 1), the two systems were generally set in place for the second and third rotation cycles. However, this avoided further improvement of the system, as we will discuss later. Finally, our experimental layout entailed only temporal repetitions (each crop is repeated every year) without spatial replication for a given year, unlike Davis et al. [8], Giuliano et al. [27] and Snapp et al. [54], whose experimental layouts were based on temporal and randomized spatial replication with much smaller fields than in Rouffach, The lack of spatial repetition lowered the statistical power of the results, although spatial soil diversity was limited and not controllable. However, the six-year length made it possible to tackle interannual variability, which provides a certain robustness to the results since the field size avoided a boundary effect.

With respect to the previous point, the degree of participation from stakeholders – and especially farmers – is a key issue. Participatory approaches have long been recognized as a major lever to increase the implementation of “sustainable solutions” by stakeholders [55]. We designed our cropping system using a participatory approach based on a formalized approach [9,30]. During the cropping year, the experiment was conducted by the scientific team working in conjunction with the farm manager. The management strategy and decision rules were discussed by the steering committee based on each year's results. This led to some aspects of the innovative system being adapted during first production cycle not covered by this article. The whole assessment was conducted by the scientific team, who proposed the indicators to assess the objectives set by the steering committee, and thresholds were discussed together. Nevertheless, this was not a fully participatory approach, where even assessment criteria and indicators are determined by stakeholders, as advocated by Meynard et al. [56]. For the implementation of MASC, the method was presented and discussed with the steering committee, and the decision was made to keep the initial decision rules and weightings.

While profitability, workload, energy consumption and pesticide use are assessed in many experiments [57], measured criteria like soil compaction, biological activity through soil microbial biomass, enzymatic activity and grain quality and calculated indicators such as those for pesticide transfer or nitrogen emissions are a novel feature of our experiment. Some of the experiments in the review by Deytieux et al. [57] looked at phosphorus, but most focused on soil organic matter and biological aspects were even not mentioned. But unlike Davis et al. [8] and Giuliano et al. [27], we assess weed density only by visual observation rather than by counting. Despite the importance of the topic as mentioned above, the work required exceeded the means allocated to the experiment and the period of counting conflicted with management issues (need for weed control) as noted by Lechenet et al. [12] for prospective experiments. Even without quantitative data, the qualitative observations were sufficient to highlight several weeding problems. For pesticide transfer, we assessed the transfer risk to groundwater for the assessment of objective achievement and transfer to surface water and air. Davis et al. [8] used another model that was more comprehensive and quantitative but also less sensitive to soil and management factors, while Giuliano et al. [27] carried out measurements of pesticide transfer to groundwater. Finally, as pointed out by Deytieux et al. [57] in their review of system experiments, no assessments of associated biodiversity (unmanaged flora and fauna) were carried out. This is due to the investment for field measurements and the lack of predictive indicators based on model outputs [58], although the qualitative indicator from DEXiPM could be used (see Ref. [26]).

Assessing whether objectives are achieved may raise questions about how these objectives are determined. While Davis et al. [8] and Snapp et al. [54] did not included an explicit assessment of objectives, instead limiting their work to a statistical comparison of systems, Giuliano et al. [27] established some explicit relative references for the innovative systems with regards to the reference system (e.g., yield of diversified system = 0.90 yield of reference system). We established a quantified explicit objective for nitrate leaching, pesticide transfer to groundwater and energy consumption (Table 2), while for other objectives, the reference was either absolute (workload, TFI) or only qualitative for the rest of the objectives, e.g., the innovative system should perform better than the reference system without greater precision on the magnitude of the difference (e.g., profitability, soil fertility). For fossil energy, the objective may have been quantified if the National Low-Carbon Strategy (Ministère de l’Agriculture et de l’Alimentation, 2019) had existed at the beginning of the study as explained in section 4.1. Regardless, this combination of reference types is common in system experiments [57].

### 4.3 Avenues for progress in innovation

The outcome of the assessment of whether objectives were achieved and the overall sustainability of the system highlighted potential room for improvement. Aside from two very low yields for wheat and soybean, a major reason the economic objective of profitability was not achieved in the innovative system was the overuse of herbicides on maize. This overuse may be attributed to reduced tillage with total elimination of ploughing. Colbach and Cordeau [50] showed that a balanced rotation without more than 30 % of one crop type (winter, spring, pluriannual), the introduction of a legume crop as well as occasional ploughing may play important roles in reducing weed pressure. The introduction of a pluriannual crop like alfalfa in a particular context would require a major reorganization of the production chain and would be possible when livestock and arable farms cohabit the same area [59]. Some possibilities exist with farms in the Vosges mountains, but such innovations go further than the scale of the cropping system studied here. A more balanced rotation with a 50/50 ratio of spring/winter crops would be progress, with a rotation that alternates both winter or spring sowing dates, which is a major factor of weed control [60]. Introducing a crop with an early spring sowing date in the rotation (e.g., spring barley) instead of only crops with a late spring sowing date (maize, soybean) would even increase the diversity. Furthermore, Colbach and Cordeau [50] also included many more management options in their study, such as tillage timing, herbicide application, type of herbicides, mechanical weeding, etc. and tried to find a compromise between yield loss reduction and ecological functions of weeds for pollinators such as butterflies. It should be noted that mechanical weeding was impaired by the presence of crop residues on the surface and was not implemented after some design failures in the first rotation cycle, but this option deserves to be comprehensively tested to identify favorable conditions for its implementation.

Occasional ploughing may be another way to reduce negative consequences of reduced tillage, although the benefits of no-till or reduced tillage are still discussed. A review by Blanco-Canqui and Wortmann [61] and a meta-analysis by Peixoto et al. [62] provided some insights, showing both positive and negative results. But long-term experiments comparing occasional tillage with ploughing and reduced tillage without ploughing are still lacking and tillage treatment for occasional tillage is not included in all experimental ploughing, making the application of results to our study more difficult. It seems that with ploughing treatments, negative effects may be observed but the number of studies remains low.

In any case, occasional tillage as well as mechanical weeding would contribute to increased fossil energy consumption. Yet this objective is not totally achieved, which means that additional efforts must be made. A major avenue for progress would be substantially reducing mineral fertilizer use, as this is the major source of energy consumption [41]. In a specific meta-analysis on maize, Wei et al. [63] showed an overall reduction in the environmental impact linked to fertilizer use when replacing mineral fertilizer with an organic product in studies focusing on emissions after application. However, when considering the entire life cycle, including production, processing, transport and application, the picture is not as clear [64]. One key way to reduce fertilizer would be to introduce more legumes in the rotation, but the rate should not be overly high to avoid problems with disease [65]. The rate of 33 % is probably a maximum value. Thus, a higher degree of diversification of the rotation, including the introduction of multispecies cover crops, is an important lever to reduce environmental impacts and improve weed control (although economic consequences should be closely assessed).

As an alternative to maize monoculture, a three-year rotation (maize-soybean-winter wheat or a four-year crop rotation (maize-maize-soybean-winter wheat like in Snapp et al. [54]) have been proposed. Yet sowing a cover crop after maize (and even after soybean) is not an easy task, especially in temperate areas with cold winters; undersowing deserves much more technical investigation to find a practicable solution. Another sequence, maize-winter barley + soybean (where “-” represents the change of year and “+” the multiple cropping), was also tested in an experiment associated with our study, but it showed very mixed results with many technical difficulties due in part to limited climatic windows for the second crop (results not shown). More recently in an *ex ante* assessment study (before implementing and testing in a system experiment), the alternative system to maize monoculture was more complex with multiple cropping but without undersowing in maize: (rye/faba beans)-maize-winter barley + soybean-winter wheat + sorghum, where “-” represents the change of year, () the cover crop and + the multiple cropping [66].

## 5 Conclusion

The aim of this study was to fill the knowledge gap on the sustainability performance of innovative cropping systems as alternatives to maize grown in irrigated continuous rotations, a highly intensive system that causes a variety of concerning environmental impacts. Following an initial three-year period to adapt the design of the innovative system, the assessment of the stabilized systems over six years showed a substantial improvement in the overall sustainability of the innovative system, which was designed with a three-year rotation of maize/soybean/winter wheat (plus a cover crop) combined with reduced tillage. Clear progress in the environmental performance and a high level of stability for the social dimension were achieved, but the economic performance fell when different price scenarios were considered. The environmental assessment included classic criteria such as energy consumption, pesticide use and nitrate leaching along with more original criteria such as pesticide transfer to groundwater, soil compaction and biological activity. Major drawbacks of the innovative system were lower profitability when scenarios of high maize prices were included, high herbicide use in maize and herbicide transfer risk to groundwater. The main reasons may be the context of the site, which has a high weed density, and the introduction of reduced tillage in the innovative system. As a result, this type of system experiment should be repeated in other contexts, including in fields with low weed density. More diversified rotations with multiple cropping and higher rotation lengths may provide additional innovation, although the possibilities in areas with cold winters are more limited than in warmer climates. Finally, because this experiment was conducted mainly by a scientific team, another area to explore would be to get stakeholders and farmers involved at the assessment phase that we have presented here in addition to having them participate in the system design phase. In particular, scenarios on upscaling the studied cropping systems at regional level should be assessed with stakeholders to determine their impacts on the agrifood chain.

## Data availability

The datasets generated during and/or analyzed during the current study are available from the corresponding author on reasonable request.

## CRediT authorship contribution statement

**Christian Bockstaller:** Writing – review & editing, Writing – original draft, Formal analysis. **Aimé Blatz:** Formal analysis, Data curation. **Olivier Rapp:** Formal analysis, Data curation. **Rémi Koller:** Writing – review & editing, Funding acquisition, Formal analysis, Data curation. **Sophie Slezack:** Writing – review & editing, Writing – original draft, Formal analysis, Data curation. **Anne Schaub:** Writing – review & editing, Funding acquisition, Formal analysis, Data curation.

## Declaration of competing interest

The authors declare that they have no known competing financial interests or personal relationships that could have appeared to influence the work reported in this paper.
