# Supplementary material for: Sustainable alternative to irrigated maize monoculture in a maize-dominated cropped area: Lessons learned from a system experiment
Source: Heliyon. 2024 Apr 26;10(10):e30400. doi: 10.1016/j.heliyon.2024.e30400 (PMC11103431; doi:10.1016/j.heliyon.2024.e30400)
Supplement: Multimedia component 1 [file mmc1.docx]

**Supplementary material**

**Sustainable alternative to irrigated maize monoculture in a maize-dominated cropped area: Lessons learned from a system experiment**

Christian Bockstaller^1*^, Aimé Blatz^1^, Olivier Rapp^2,3^, Rémi Koller^2^, Sophie Slezack^4^, Anne Schaub^5^

^1^Université Lorraine, INRAE, LAE, F-68000 Colmar, France

^2^Association pour la Relance Agronomique en Alsace (ARAA), Schiltigheim, F-67013 Strasbourg, France

^3^Chambre d’Agriculture Alsace, 67500 F-Haguenau, France

^4^Université Lorraine, INRAE, LAE, F-54000, Nancy, F-France

^5^Chambre Régionale d’Agriculture du Grand Est (CRAGE), Schiltigheim, 67013 F-Strasbourg, France

*Corresponding author: christian.bockstaller@inrae.fr

**Supplementary material 1: Data on production situation, soils and experimental layout**

1. **Socioeconomic environment of the supply chain**

The maize supply chain makes up a significant share of agriculture production in the Alsace region, accounting for 58% of the cropped area, whose 91% is grain maize (Agreste 2020). About 40% of the total production is intended for the food industry (starch manufacturers and semolina mills), with more than half processed in Alsace (Agreste 2020). The remaining 60% is exported from Alsace to neighboring industries along the Rhine River. After maize, winter wheat is the second most-produced crop; it accounts for 20% of the cropped area, with recent trends moving upward. A non-GMO soybean supply chain for food production has also recently emerged and is growing, totaling 1% of the cropped area (1640 ha).

1. **Biotic pressure in the cropped area**

The main crop pests for maize and soybean are weeds, and specifically field bindweed (*Convolvulus arvensis* L*.*), lamb’s quarters (*Chenopodium album* L.) and summer grass species, primarily *Echinochloa crus-galli* (L.) P.Beauv. and *Setaria verticillata* (L.) P.Beauv. The European corn borer (*Ostrinia nubilalis*) is the main insect pest for these crops, and pressure from this pest is high in the cropped area. Soils pests, including wireworms (*Agriotes* spp. and *Athous* spp.) and the corn rootworm (*Diabrotica virgifera virgifera*), may also cause significant damage. Other pests such as Corvidae consume seeds before emergence or seedlings and may cause considerable damage; in the worst cases, farmers may have to resow the field. For wheat, the main diseases are *Septoria* leaf spot (*Mycosphaerella graminicola*) and *Fusarium* head blight (*Fusarium* *spp.*). Slug damage can occur at sowing when spring weather is cold and wet. Finally, mammals such as boars (*Sus scrofa*) and hares (*Lepus europaeus*) may harm crops (there is a forest within 250 m of the experimental fields).

1. **Data on soils (see Table S1)**

Table S1: Additional soil characteristicts: a) soil description (2002), b) chemical composition (2009, 2015, 2018) with double analysis due to stratification in the innovative system (INN) due to reduced tillage.

a)

b)

1. **Experimental layout**

Fig. S1: Google Earth view of the system experiment. The reference system is located on the plot outlined in solid line and the innovative system on the plots outlined in dotted line.

**Supplementary material 2: example of decision rules for weed control strategy**

Fig. S2: Detailed strategy for weed control

**Supplementary material 3: Calculation of indicators to evaluate the economic, social and environmental objective**

1. **Profitability**

Profitability was assessed by the calculation of a semi-net margin:

**production + CAP subsidies − variable costs − mechanization costs** (Equation S1)

where:

Production (euros/ha) is the normalized yield (t/ha) multiplied by the price (euros/t) and divided by the field area (ha).

CAP subsidies: The average subsidies from the common agriculture policy for the Haut-Rhin *département* (364 euros/ha in 2013) are included in the calculation while the drying costs of maize are directly deducted from the price.

Variable costs (in euros) include seeds, fertilizers, pesticides and adjuvants, and irrigation costs.

Mechanization costs refer to equipment costs that can be attributed to the crop according to a French database that indicates the cost of fuel and equipment maintenance and depreciation based on use (Bureau de Coordination du Machinisme Agricole 2012).

The semi-net margin was calculated for eight contrasting real-price scenarios as in Lechenet et al. (2014) to assess the effect of price volatility. The price scenarios integrated harvested crop and fertilizer prices and were based on real data between 2007 and 2014 (Table S3). Scenario 1 used for the multicriteria assessment was based on medium output and input prices, as was scenario 4. Two scenarios (7 and 8) used low output and input prices, one scenario (6) low output and high input prices, one scenario (2) medium output and low input prices, one scenario (5) medium output prices and high input prices, and one scenario (3) high output and medium input prices (see Table S3).

Table S3: scenario prices for crop outputs and fertilizers and fuel (Massot et al. 2016)

^a^ excepted for maize

^b^ excepted for fuel

^c^ excepted for potash

1. **Workload**

Workload was calculated by assessing the total duration of each field operation by means of the CRITER 5.4 software (Craheix 2015). Detailed software outputs make it possible to calculate the total workload (hours/ha/year) as well as the distribution throughout the year to identify workload peaks in an Excel file.

1. **Reduction of environmental impacts**

Soil fertility was assessed by monitoring chemical soil properties at the start (2009) and end (2018) of the experiment. Soil organic matter and main nutrient contents (P, K) measured on soil samples from the tilled layer at depths of 0–10 cm and 10–25 cm according to standard laboratory protocol. Physical fertility through soil structure was assessed visually by the method described in Roger-Estrade et al. (2004) (Supplementary Material 4). Soil microbial enzyme activities and extractable soil C and N were assessed according to Romillac et al. 2015 (Supplementary Material 11).

Pesticide reduction is addressed by the treatment frequency index (TFI), (Gravesen, 2003), currently used in the French national pesticide reduction plan (Ecophyto) and is calculated as described in Lechenet et al. (2014). This indicator is calculated for each pesticide application as follows:

Application rate x| Treated surface area

TFI = ^___________________________________________________^  (Equation S2)

Registered dose x Plot surface area

where the application rate and the registered dose are both expressed for a given commercial product (which may contain several active ingredients). The registered application was the lowest registered application dose if the pesticide can be applied for several target pests. The TFI for a given crop season was then calculated as the sum of the TFI for each pesticide application performed during the crop season.

Pesticide transfer to groundwater, surface water and air was assessed by I-Phy3 (v1.70) from the INDIGO method (Pierlot et al. 2023, see Supplementary Material 5). We implemented three I-Phy3 sub-indicators: pesticide leaching (RCgw_lea_), pesticide transfer by runoff (RCsw_ru/d_) and pesticide volatilization (RCair_Vol_), without considering toxicity on targeted organisms. For drinking water, stakeholders are only concerned with the pesticide concentration based on the water quality standard of 0.1 µg/L.

For nitrate leaching, we used the nitrogen indicator (I-N v.2.70) from the INDIGO method (Bockstaller et al. 2008, see Supplementary Material 6). I-N v.2.70 is based on an operational model that assesses nitrate leaching (I-NO_3_) and nitrogen gaseous emissions (NH_3_ and N_2_O) in a quantitative way by using a reduced number of available input data, average weather data, simplified soil descriptions (texture and depth classes, soil organic matter in the tilled layer, etc.), and crop and fertilizer management data. Several complex inputs, such as a complete soil profile description or wind speed (which is a relevant variable for NH_3_ volatilization), are not included in the calculation.

Energy consumption was calculated using the I-En indicator from the INDIGO method (Pervanchon et al. 2002). It calculates direct energy consumption for the main consuming management component (machinery traffic and irrigation) and indirect consumption for the main consuming component (fertilizer and pesticide production). Updated energetic coefficients for fertilizer were taken from Arvalis-Institut du Végétal et al. (2020).

The semi-net margin and TFI were calculated using the CRITER 5.4 software (Craheix 2015), while I-Phy, I-NO_3_, I and I-En indicators were calculated using specific Excel sheet calculators.

**Supplementary material 4: Assessment of physical fertility of soil inspired from the method of Roger-Estrade et al. (2004)**

**Methods**

A pit has to be dug in an area of the field which is representative of the spatial variation of soil structure induced by traffic and tillage.

The observed ace of the pit is then described by a double stratification:

- A vertical one to identify different horizons: impacted by different field operations (from seedbed to the horizon impacted by former ploughing.
- A horizontal to identify wheel tracks (tillage, other field operations) and area not impacted by traffic.
- Thus compartments are defined on the observation face of the pit by the intersection of the horizons and the location of the wheel tracks

Each compartment is then visually observed with two criteria:

- The degree of compaction: here in four classes
- The morphological aspect of clods is classified in three classes:
  - Γ: clods with highly visible porosity (favourable)
  - Φ: clods with cracks due to weathering
  - Δ: clods with no visible porosity (unfavourable)

We used decisions rules shown in table S4 to aggregate both criteria.

Table S4: Decision rules to assess soil structure from two criteria: morphological aspect of clods and degree of compaction and colour used for the representation of results.

| Morphological aspect of clods/  Degree of compaction | **Γ** | **Φ** | **Δ** | **Hydromorphe area** |
| --- | --- | --- | --- | --- |
| Fine | **Favourable** | **Favourable** |  |  |
| Half fine | **Very favourable** | **Rather favourable** | **Funciton of clods size** | **Very unfavourable** |
| Half dense | **Rather favourable** | **Slightly favourable** | **Unfavourable** | **Very unfavourable** |
|  |  |  | **Very unfavourable** | **Highly unfavourable** |
| Massive |  | **Unfavourable** |  |  |
|  |  |  | **Highly unfavourable** |  |

**Supplementary material 5: Description of the component of I-Phy indicator and its subcomponents**

I-Phy3 is an indicator of the INDIGO method (Bockstaller et al., 2008; Lindahl and Bockstaller, 2012) which is structured in three levels of aggregation as shown on Fig. S5.1 below. The global indicator I-Phy3 results from the aggregation of three risk indicators tackling three environmental indicators like in version 1 (van der Werf and Zimmer 1998): groundwater, surface water and atmosphere. At the bottom, five basic subindicators assesses the risk of contamination (RC) which is a major issue of concern for many stakeholders due to the environmental standards for example for water which rests on concentration and not on risk level. Pesticide leaching determines groundwater contamination (RCgw_lea_) while surface water may be contaminated by pesticide runoff or drainage transfer (RCsw_ru/d_) or pesticide drift (RCsw_dr). Air contamination is due to pesticide volatilization (RCair_vol_) and/or pesticide drift (RCair_dr_). Subindicators RCgw_lea_, RCsw_ru/d_, RCair_vol_ are used in the assessment of global sustainability with the MASC model (Craheix et al., 2016).

The indicator I-Phy3 as well as all subindicators (1^st^ and 2^nd^ levels, see Fig. S5.1) are expressed on an performance scale between 0 (high risk) and 10 (low riks), (Bockstaller et al., 2008). The five risk of contamination are calculated for 1 kg of active ingredient and modulated by the effective rate. The method proposed by Lindahl and Bockstaller (2012) is used to calculate an effective rate of a.i. available for transfer. the initial rate being weighted by the interception rate. They designed an algorithm which makes possible to reduce or increase the indicator value obtained for 1 kg a.i., in function of this effective rate. Last each of these five subindicators is transformed in a risk between 0 (no) and 1(high) and weighted by the percentage of sprayed area, and then retransformed in a value of performance. We assumed that there is a proportional relation between sprayed area and level of contamination (Melland et al. 2015).

Fig. S51: Overview of the calculation of I-Phy3 with three levels of aggregation and main input variables, in blue pesticide properties, in red soil and topographic variables, in green : management variable (KOC: adsorption coefficient, DT50: half life, Solubility: solubility in water, Ps: vapor pressure, LogKow: logarithm of the octanoal-water coefficient, time: time between spraying and runoff event, ADI: admissible daily intake, Aquatox: highest toxicity between fish, daphinae and alguae.

Fig. S5.2 shows the decision tree for calculating RCgw_lea_. In comparison with initial version of I-Phy (van der Werf and Zimmer 1998), RCgw_lea_, includes an additional property: solubility and the period of application, spraying on wet soil in winter increasing the transfer risk (Pierlot et al. 2017). In this new version, pesticides leaching potential also tackles the effect of no-tillage which increases pesticide leaching (Alletto et al. 2010; Dairon et al. 2017).

Fig. S5.2: Decision tree for the calculation of the risks of groundwater contamination by leaching (RCgw_lea_) for favourable (white) and unfavourable (grey) cases. Intermediate cases are covered with fuzzy logic (Pierlot et al., 2023).

Fig. S5.3 shows the decision tree for calculating RCsw_r/d_. This results from the aggregation of a runoff potential already used by van der Werf and Zimmer (1998), and a variable assessing the availability of pesticide based on equation S3. The runoff potential includes the effect of tillage that was newly parametrized with data from the metanalysis of Elias et al. (2018).

**Ava = e^-Ln(2)/(DT50.t)^**       (Equation S3)

Where :

Ava : availability of active ingredient

DT50 : soil half life of active ingredient (days)

t : time in days between date of spraying and date of next runoff event with a default value of 3 for a worst case situation (Pierlot et al., 2017).

RCair_vol_ was totally changed with respect to the calculation method of van der Werf and Zimmer (1998). It is based on empirical equations of Woodrow et al. (1997) calculating an volatilization flow on soil function of vapor pressure, solubility and the adsorption coefficient KOC) and on plant function of vapor pressure. Volatilization flow from soil is weighted by an abattment factor taking into account the effect on plant residue (assessed with tillage category) and volatilization flow from plan is weighted by a factor addressing role of formulation and the effect of the logarithm of the octanoal-water coefficient (LogKow ). The total volatilisation may be reduced if field margin consists in a dense hedge with sufficient height.

Data on the predictive quality of RCgw_lea,_ RCsw_r/d_. and RCair_vol_ can be found in Pierlot et al. (2023). Their correlation with measured data are at the expected level for such type of indicator.

Fig. S5.3: Decision tree for the calculation of the risks of surface water contamination by runoff (RCsw_r/d_) for favourable (white) and unfavourable (grey) cases. Intermediate cases are covered with fuzzy logic (Pierlot et al., 2023).

**Supplementary material 6: description of the Nitrogen Indictor (I-N v.2.70)**

The nitrogen indicator belongs to the INDIGO method (Bockstaller et al., 2009; 2008) and rests on three subindicators: I-NO3, I-NH3, I-NO2 addressing respectively nitrate leaching, ammonia and nitrous oxide emissions. Those are calculated by an operational models in a quantitative way. Concept of operational model refers here to the choice of input variable based on the availability of data. Several complex inputs such as the wind speed which is a relevant variable for NH3 volatilisation are not included into the nitrogen indicator IN. Nitrate leaching is not calculated by simulating the whole nitrogen cycle but at key period as stated by (Durand et al. 2002), (Fig. S6), after fertilization and after harvest during the drainage phase. Bot results from :

- the calculation of a leaching factor with the simplified Burn’s equation including climatic data for drainage and soil data for volumetric field water capacity.
- The calculation of an amount of available nitrogen in soil.
  - For leaching after fertilization, it is the amount of nitrogen in fertilizer weighted by an availability factor. This factor is function of the date of fertilization. When nitrogen is applied during the period of intensive growth, it is zero and the earlier the nitrogen is brought before this period, i.e. the interval between fertilization and intensive growth is higher, the higher is the factor until to reach 1, i.e. the whole amount of fertilizer is available for leaching.
  - For leaching after harvest, a mineral nitrogen balance in the system soil-plant (Oenema et al. 2003) where mineralization of crop residues and mineralization are the main inputs and nitrogen uptake by a crop or a catch crop before drainage the main output.

Ammoniac volatilization is calculated by an emission coefficient factor of the Total ammonia nitrogen amount. The volatilization factor were retrieved from literature for different fertilizers and depend on fertilization period (temperature effect) and incorporation (management effect with a reduction of 50% of volatilization).

Nitrous oxide is based on the (Bouwman 1996) factor (0.0125) weighted by sereval factors:

- An additional factor (0.0175) is applied to the overfertilization (Germon et al. 1999) because it increases soil nitrogen at harvest (ten Berge 2002).
- Soil characteristics, hydromorphic or peat soil increase the emission.
- Irrigation

The effect of reduction is not considered according to (van Kessel et al. 2013)

Calculation of each component is detailed in Table S6

In a second step model outputs are transformed into scores between 0 (high leaching) and 10 (no losses) with a value of 7 meeting environmental standards (e.g. 50 mg NO_3_/L in water) and may aggregated in a single score by a law of minimum.

Fig. S6: Overview of the Nitrogen Indicator **(I-N v.2.70)** assessing nitrogen losses, nitrate leaching, ammoniac and nitrous oxide emission. The main input variables involved in the different nitrogen losses are given in the boxes. Blue arrays and red arrays represent respectively the nitrogen inputs and the evolution of nitrogen in the agroecosystem, leading to gaseous emission, plant uptake, and leaching to groundwater (SMN= soil mineral nitrogen).

Data on the predictive quality of I-NO3 can be found in Bockstaller et al. (2022) under the abbreviation I-N2.. It yielded mitigated results better than another model but worse than the more elaborated version 3 of I-N..

Table S6: Calculation of components of I-N. Values are in kg N/ha excepted value lower than 1 or with another unit

| Component | Explication | Example |
| --- | --- | --- |
| I-NO3 | **I-NO3 = INO3f + INO3h**  Where  I-NO3: nitrate leaching (kg NO3-N)  I-NO3f : nitrate leaching (kg NO3-N) after fertilization  I-NO3h: nitrate leaching (kg NO3-N) after harvest |  |
| Leaching factor (LF) | Simplified Burn’s equation  **LF = (D/(D=V/10)^z^**  Where  D: drainage (mm)  V: volumetric water field capacity  z: depth factor | Sandy soil : V=0.15, silty soil: V=0.30, clayly soil V=0.45  z = soil depth (cm) if nitrogen at surface (after fertilization)  z = soil depth/2 if nitrogen in soil profile is homogeneous (after harvest) |
| I-NO3 after fertilization | **INO3f = Nf . LF . a**  Where  N_f_: fertilizer rate  a: avaiability factor N  **a = (di-df)/(di-d1)**  Where  df: fertilisation date  di: date of intensive growth  d1: date where a = 1 | Winter wheat, winter rapeseed  di = d1+ 110 d1 = January 1^st^  Sugar beet  di = d1+ 100 d1 = sowing date  Maize  di = d1+ 80 d1 = sowing date |

Table S6: Calculation of components of I-N (cont.)

| Component | Explication | Example |
| --- | --- | --- |
| I-NO3 after harvest | **I-NO3h = SMNdr . LF**  Where  SMNdr = soil mineral nitrogen at beginning of drainage  **SMNdr = SMNh + Nminres + Nminsoil + Xh – Nccup -Ncup**  Where  SMNh : soil mineral nitrogen at harvest  Nminres: mineralization (or immobilisation) due to crop residues  Nminsol: mineralization of soil organic matter after harvest  Nccup and Ncup: nitrogen utpaken by catch crop (cc) or crop (c) before drainage:  **SMNh= f (X-X_R_)**  Where  X= fertilizer rate  X_R_= recommended rate  **Nminsoil = Nminsoil_y_*Ic**  Where  Nminsoil_y_: yearly soil mineralization of organic matter (Taureau et al. 1996)  Ic: faction of Nminsoil_y_ not uptaken by crop | SMN at harvest = minimum value if X-X_R_ ≤ 0  SMN at harvest = 0.5 . (X-X_R_)  if X-X_R_ > 0  Nminres: =20 for winter rapeseed  = 0 for cereal straw (removed)  = -20 for wheat straw (ploughed in)  = for maize straw  = 20 for sugarbeet  Ic for winter rapeseed = 0.60  Ic for winter wheat = 0.50  Ic for maize = 0.30  Ic for sugar beet = 0  Nccup = 10-80  Ncup for winterrapeseed = 10-100  Ncup for winterwheat=5-35  Ncup for maize, sugar beet = 0 |
| I-NH3 | **I-NH3 = TAN * c_NH3_**  Where  I_NH3_ = Ammoniac volatilization  TAN : total ammoniac nitrogen  c_NH3_: volatilization factor | For mineral fertilizer, TAN = nitrogen content*rate  For organic fertilizer: TAN = mineral nitrogen content * rate  In summer without incorporation, soil rich in limestone  For ammonium nitrate c_NH3_ = 0.3  For diammonium phosphate  c_NH3_= 0.15  For N solution c_NH3_ = 0.15  For urea c_NH3_ = 0.18 |
| I-N2O | **I-N20 = X . c_N2O_ . ci**  Where  I-N2O = Ammoniac volatilization  TAN : total ammoniac nitrogen  c_NZO_: emission factor  ci: correction factor | c_N2O_= 0.0125 if X-XR ≤0  Additional term: 0.0175 . (X-XR)  if X-XR >0  For peat soil ci = 2  If only hydromorphic ci = 1.25  If irrigation = 1.20  Different correction factor are multiplied |

**Supplementary material 7: Agronomic assessments**

Table S7: overview of methods to assess component of the agronomic assessment

| Theme | Subtheme | Method | Detail |
| --- | --- | --- | --- |
| Maize grain yield |  | Measurement | By combine harvester |
| Soybean yield |  | Measurement | By combine harvester |
| Wheat yield |  | Measurement | By combine harvester |
| Grain quality | Maize grain mycotoxins | Measurement | Protocol of accredited laboratory |
|  | Wheat grain mycotoxins | Measurement | Protocol of accredited laboratory |
|  | Wheat protein content | Measurement | Standard protocol of accredited laboratory for N analysis (Dumas method)^1^. Protein = 5,7 x N |
| Weed control |  | Visual assessment | Once a week from seed to harvest |
| Pest control |  | Visual assessment and counting | Maize before harvest : counting in 6 x 10 m  Visual : once a week |
| Disease control |  | Visual assessment and counting | Maize before harvest : counting in 6 x 10 m  Visual : once a week |
| Water supply |  | Calculation of water balance |  |
| Nitrogen supply |  | Visual assessment |  |
| Nitrogen uptake by cover crop after wheat |  | Measurement | Measurement of fresh and dry biomass in 9 x 1 m². Separation of different species.  Analysis of N by standard protocol of accredited laboratory |

^1^<https://tools.cofrac.fr/fr/organismes/fiche.php?entite_id=12082539>

**Supplementary material 8: The MASC method**

Finally, we performed a multicriteria assessment of sustainability by means of the MASC 2.0 method (Craheix et al. 2016) supported by the DEXi software (Bohanec et al. 2008). Through this formalism, MASC conceptualizes the sustainability assessment problem by breaking it down into the three typical dimensions used to define sustainability: social, economic and environmental. For each dimension, indicators are organized into a tree-like structure (decision tree) created using 38 basic indicators and 26 aggregated indicators. All the indicators are expressed in qualitative classes (e.g., low, medium, high) after discretization of the quantitative indicators. Class limits are assigned based on the method or were adapted to the regional context (such as the semi-net margin). Aggregation rules consist of linguistic rules expressed in “if–then” decision rules determined by the users or automatically by the DEXi software according to user-specified weighting. Here, we kept the aggregation rules established by the authors of the MASC method. Indicators used to assess the achievement objectives assigned to the cropping systems belong to the set of 38 basic indictors (e.g., semi-net margin, I-N v.2.70 and I-Phy indicators). Most of the basic indicators are calculated by the CRITER 5.4 software (Craheix 2015). Results are expressed as a synoptic table representing the decision tree, from the basic indicators through the different aggregation levels and finally to the global sustainability indicator (see Fig. 4). Details on the basic indicators are given in Table S8.

Table S8 Multicriteria assessment with help of the MASC method. Dimension Ec: economic, S: social, En: environmental. Underlined variable in the column “calculation method” refers to a basic MASC indicator. More details can be found in Craheix et al. (2011).

| Dimension | Basic MASC indicator | Calculation method |
| --- | --- | --- |
| Ec | Profitability | =f (Semi-net margin) (see section 2.5.1 |
| Ec | Economic independence | =f (Ratio total subsidies/semi net margin) |
| Ec | Economic efficiency | =f (Total output/operational and mechanisation costs) |
| Ec | Soil acidity state control | Assessment by soil analysis |
| Ec | Soil structure control | Assessment by observations in soil pits and by feeling of farmer and experimenter |
| Ec | Phophosrus and potassium fertility control | Assessment by soil analysis |
| Ec | Weed control | Assessment by observations |
| Ec | Pest control | Assessment by observations |
| Ec | Sanitary quality | Assessment by mycotoxin analysis |
| Ec | Technical and visual quality | Assessment by observations and protein analysis |
| Ec | Contribution to emergence of new activity | Assessment by local expertise |
| Ec | Contribution to local employment | =f (working time of seasonal workers) |
| S | Raw material supply | =f (innovative yield/mainstream yield) |
| S | Complexity of management | =f (crop type). e.g; winter cereals =1, non irrigated maize = 1, irrigated maize =2, winter oilseed rape = 2, sugar beet = 3 |
| S | Scouting time | =f (time) |
| S | Work overload | Assessment by local expertise |
| S | Risk of farmer health | =f (number of spraying with at least 1 pesticide classified Xn: hazardous, T: toxic or T+: very toxic) |
| S | Physical difficulties | =f (number of physical constraints) |

Table S8 (Cont.)

| Dimension | Basic MASC indicator | Calculation method |
| --- | --- | --- |
| Env | Pesticide groundwater | See SM5: I-Phy3 |
| Env | Pesticide surface water | See SM5: I-Phy3 |
| Env | NO_3_ loss control | See SM6: I-N |
| Env | P loss control | DEXi model =f (erosion control, P content in soil, P rate, Incorporation) |
| Env | NH_3_ emission control | See SM6: I-N |
| Env | N_2_O Emission control | See SM6: I-N |
| Env | Pesticide emission to air control | See SM5: I-Phy3 |
| Env | Control of accumulation of toxic substances | Assessment by expertise |
| Env | Soil organic control | =f (soil organic matter indicator of INDIGO method (Bockstaller et al. 1997)) |
| Env | Erosion control | Assessment by observations |
| Env | Water use of irrigation during critical period | Assessment of water use and critical period by local expertise |
| Env | Dependency on water resource | DEXi model =f (Crop water requirement, resource autonomy) |
| Env | Energy use | =f (Energy indicator of INDIGO method (Pervanchon et al. 2002)) |
| Env | Energetic efficiency | =f (energy in outputs/energy use) |
| Env | Phosphorus pressure | =f (non-renewable P use) |
| Env | Conservation of flying insects | DEXi model = f crop family diversity, TFI insecticide) |
| Env | Conservation of soil macrofauna | DEXi model = f (tillage, organic matter supply, TFI insecticide) |
| Env | Flora abundance | Weed control |
| Env | Flora diversity | Assessment by observations |
| Env | Conservation of micro-organisms | DEXi model = f (organic matter supply, TFI, crop family diversity) |

**Supplementary material 9: Biology activity**

**Introduction**

Crops, in combination with agricultural practices, may alter soil biological activity and nutrients availability. It is commonly reported that integration of legumes into cereal-based rotations may improve the availability of soil N, with positive impacts on the supply of succeeding crops and on yield (Arcand et al., 2013), as observed for example by comparing maize in monoculture or in rotation with legumes (Uzoh et al., 2019). These effects are related to the alteration of soil properties induced by plants in combination with agricultural practices and in particular, soil organic C and N pools. These pools, depending on their quantity and quality, are assumed to play a critical role in soil microbial enzymatic activities that govern the decomposition/mineralization processes (Sinsabaugh et al., 2008). As these microbial enzymes, as well as other biological variables such as microbial biomass, react sensitively and rapidly, they have been widely used to monitor temporal changes in soils following changes in cropping systems and agricultural practices. We compared the effects of maize in monoculture with ploughing tillage and maize cultivated in crop rotation with winter wheat and soybean on soil microbial activities implicated in N cycling and other variables related to C and N availability which are assumed to be drivers of these microbial activities, over the three years of the rotation.

**Material and methods**

***Soil sampling***

Soil samples were collected in each experimental field in 3 plots (with 5 soil cores per plot), one time a year (in April – May), from 2014 to 2016 (6 to 8 years after field-experiment establishment). Soil cores were sampled (0-15 cm, 5 cm in diameter) in each plot, thoroughly homogenized and were then sieved at 5 mm, and stored at 4°C until use (less than one week).

***Soil analyses***

*Soil microbial biomasses*

Soil microbial biomass C and N were determined using the fumigation-extraction method (Vance et al., 1987). The chloroform-fumigated and non-fumigated extracts in 0.5M K_2_SO_4_ (1:5 w/v ratio) were frozen at -20°C until analysis. The concentration of extractible C and N in soil extracts were determined using a TOC-V CSH (Shimadzu). Conversion factors of 0.45 (Joergensen, 1996) and 0.54 (Brookes et al. 1985) were used to convert extractable C and N into microbial biomass C and N (expressed in mg C or N per kg of soil).

*Soil extractable C and N*

The C and N in the K_2_SO_4_ extracts from non-fumigated samples were determined using a TOC-V CSH (Shimadzu) and used to estimate extractable organic C and N (Bell et al., 2015).

*Microbial enzyme activities*

The potential soil enzyme activities of leucine aminopeptidase (LAP, EC 3.4.11.1), protease (PROT, EC 3.4.2.21–24) was determined spectrophotometrically, according to Ladd and Butler (1972). Potential enzyme activities were assessed using 2mM leucine-p-nitroanilide and 2% sodium caseinate, as substrate for LAP and PROT, respectively. Ammonification activity and nitrification activity were measured as described in Romillac et al. (2015), using arginine and ammonium as substrate, respectively.

***Data analysis***

R software (R Core Team 2013) was used for all the statistical analyses. Exploratory analysis of the data was performed using Principal Component Analysis (PCA).

**Results and discussion**

**
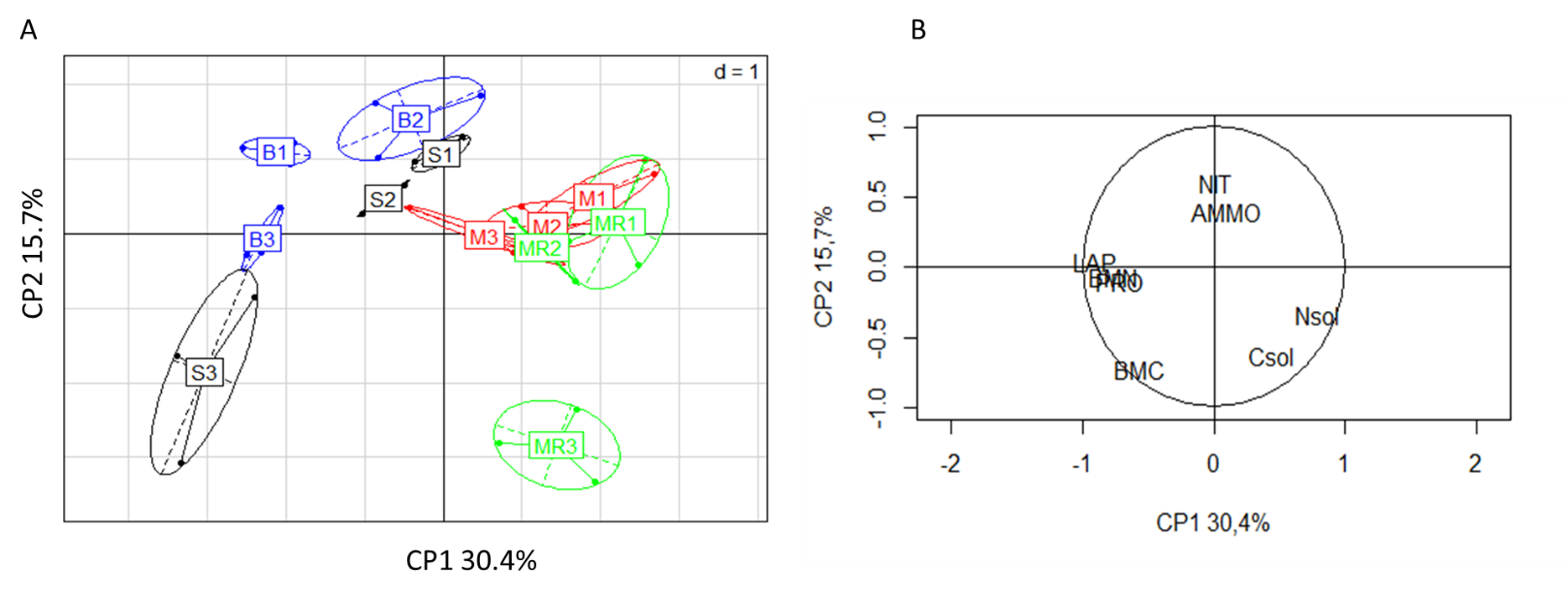
**

**Fig. S9.** Principal Component Analysis (PCA) of soil samples under maize in monoculture (M), maize in rotation (MR) with soybean (S) and wheat (W), during the three years (1, 2 and 3) of the experimentation. The following variables were analyzed: PRO, LAP, ammonification, nitrification, extractable organic C and N and C and N microbial biomass.

(A) PCA ordination of the soil samples. (B) Variables involved in the discrimination of the soil samples.

The Principal Component Analysis showed that the first and second principal axis (CP1 and CP2), explained 30.4% and 15.7% of the total variability, respectively. The PCA discriminated along PC1 soils under wheat and soybean from soils under maize, which is explained by PRO, LAP activities and N microbial biomass that tend to be higher under wheat and soybean compared to maize. It should be noticed that soil under maize in monoculture were not discriminated from soils under maize in rotation. Finally, soils were discriminated according to year of sampling, with soils under wheat and soybeans that differed from year to year, while soils under maize in rotation differed mainly between the second and the third year of sampling

As previously reported, crops and crop successions influenced soil microbial activities and the size of soil microbial biomass (Romillac et al., 2023, Soman et al., 2017). In particular, we observed that the activities of soil microbial enzymes implicated in N mineralization were higher under wheat and soybean compared to maize. These effects could be related to the organic N pools (rhizodeposits and crop residues) that return to soil under soybean that influence soil N mineralization and notably under the succeeding crop such as wheat. Although the effect of year does not appear to outweigh the effects of crops, we showed that the year of sampling had an impact on both biomass and soil microbial activities. These effects have been described previously (Romillac et al., 2023) and could interact with crop rotation and management to modulate microbial communities involved in soil nutrient dynamics.

**Supplementary material 10:**

Table S10: detailed results of the groundwater contamination risk of I-Phy3 (RCgw_lea_) expressed between 0 (high risk) and 10 (no risk), and the explaining variable (0 unfavourable, 1: favourable) to highlight active ingredients responsible for pesticide transfer to groundwater. For more details on I-Phy3 see Supplementary Material 5. REF M: reference maize INN M: innovative maize INN S: innovative soybean INN W: innovative winter wheat.

| ***Id field*** | Harvest year | Crop | Active ingredient | Rate  (kg/ha) | RCgwlea | GUS | Spraying period | Leaching potential | Solubility |
| --- | --- | --- | --- | --- | --- | --- | --- | --- | --- |
| M_RI1_2015 | 2015 | INN M | **S-metolachlor** | **1.400** | **3.6** | **0.00** | **0.39** | **0.37** | **0.98** |
| M_RI2_2014 | 2014 | INN M | **S-metolachlor** | **1.200** | **3.6** | **0.00** | **0.38** | **0.37** | **0.98** |
| M_RI2_2017 | 2017 | INN M | **S-metolachlor** | **1.500** | **3.6** | **0.00** | **0.46** | **0.37** | **0.98** |
| M_RR_2015 | 2015 | REF M | **S-metolachlor** | **1.400** | **3.7** | **0.00** | **0.39** | **0.45** | **0.98** |
| M_RR_2017 | 2017 | REF M | **S-metolachlor** | **1.109** | **3.8** | **0.00** | **0.47** | **0.45** | **0.98** |
| M_RR_2016 | 2016 | REF M | **S-metolachlor** | **1.647** | **3.9** | **0.00** | **0.60** | **0.45** | **0.98** |
| M_RI1_2018 | 2018 | INN M | **S-metolachlor** | **0.990** | **3.9** | **0.00** | **0.53** | **0.37** | **0.98** |
| S_RI2_2018 | 2018 | INN S | **S-metolachlor** | **1.094** | **3.9** | **0.00** | **0.60** | **0.37** | **0.98** |
| M_RR_2015 | 2015 | REF M | **S-metolachlor** | **0.970** | **4.1** | **0.00** | **0.58** | **0.45** | **0.98** |
| M_RR_2014 | 2014 | REF M | **S-metolachlor** | **1.128** | **4.1** | **0.00** | **0.68** | **0.45** | **0.98** |
| M_RI2_2014 | 2014 | INN M | **glyfosate** | **2.520** | **5.0** | **0.96** | **0.18** | **0.37** | **0.00** |
| M_RI1_2015 | 2015 | INN M | **glyfosate** | **2.138** | **5.7** | **0.96** | **0.44** | **0.37** | **0.00** |
| S_RI3_2014 | 2014 | INN S | **imazamox** | **0.032** | **5.8** | **0.00** | **0.62** | **0.37** | **0.00** |
| M_RI1_2018 | 2018 | INN M | **nicosulfuron** | **0.038** | **5.8** | **0.00** | **0.72** | **0.37** | **0.00** |
| M_RI3_2016 | 2016 | INN M | **glyfosate** | **0.900** | **5.8** | **0.96** | **0.26** | **0.37** | **0.00** |
| M_RR_2018 | 2018 | REF M | **nicosulfuron** | **0.028** | **5.9** | **0.00** | **0.60** | **0.45** | **0.00** |
| M_RI2_2017 | 2017 | INN M | **glyfosate** | **1.080** | **6.0** | **0.96** | **0.43** | **0.37** | **0.00** |
| M_RI2_2017 | 2017 | INN M | **nicosulfuron** | **0.026** | **6.0** | **0.00** | **0.70** | **0.37** | **0.00** |
| M_RI1_2018 | 2018 | INN M | **glyfosate** | **1.080** | **6.0** | **0.96** | **0.48** | **0.37** | **0.00** |
| M_RI1_2018 | 2018 | INN M | **glyfosate** | **1.080** | **6.0** | **0.96** | **0.48** | **0.37** | **0.00** |
| S_RI2_2015 | 2015 | INN S | **imazamox** | **0.025** | **6.0** | **0.00** | **0.69** | **0.37** | **0.00** |
| M_RI3_2013 | 2013 | INN M | **nicosulfuron** | **0.036** | **6.1** | **0.00** | **0.83** | **0.37** | **0.00** |
| M_RR_2013 | 2013 | REF M | **nicosulfuron** | **0.028** | **6.2** | **0.00** | **0.73** | **0.45** | **0.00** |
| M_RI3_2013 | 2013 | INN M | **glyfosate** | **0.900** | **6.2** | **0.96** | **0.42** | **0.37** | **0.00** |
| M_RI1_2015 | 2015 | INN M | **nicosulfuron** | **0.021** | **6.3** | **0.00** | **0.79** | **0.37** | **0.00** |
| S_RI3_2017 | 2017 | INN S | **imazamox** | **0.028** | **6.4** | **0.00** | **0.87** | **0.37** | **0.00** |
| M_RR_2013 | 2013 | REF M | **nicosulfuron** | **0.025** | **6.4** | **0.00** | **0.80** | **0.45** | **0.00** |
| S_RI2_2015 | 2015 | INN S | **imazamox** | **0.018** | **6.4** | **0.00** | **0.79** | **0.37** | **0.00** |
| M_RR_2018 | 2018 | REF M | **nicosulfuron** | **0.014** | **6.4** | **0.00** | **0.72** | **0.45** | **0.00** |
| M_RI3_2016 | 2016 | INN M | **nicosulfuron** | **0.019** | **6.4** | **0.00** | **0.83** | **0.37** | **0.00** |
| B_RI2_2013 | 2013 | INN W | **fluroxypyr** | **0.080** | **6.6** | **0.31** | **0.63** | **0.37** | **0.00** |
| S_RI3_2014 | 2014 | INN S | **glyfosate** | **0.720** | **6.7** | **0.96** | **0.47** | **0.37** | **0.00** |
| S_RI1_2016 | 2016 | INN S | **imazamox** | **0.024** | **6.7** | **0.00** | **0.95** | **0.37** | **0.00** |
| M_RR_2014 | 2014 | REF M | **nicosulfuron** | **0.014** | **6.8** | **0.00** | **0.86** | **0.45** | **0.00** |
| S_RI2_2018 | 2018 | INN S | **imazamox** | **0.019** | **6.8** | **0.00** | **0.94** | **0.37** | **0.00** |
| B_RI2_2013 | 2013 | INN W | **prochloraze** | **0.120** | **6.8** | **0.00** | **1.00** | **0.37** | **1.00** |
| M_RI2_2014 | 2014 | INN M | **nicosulfuron** | **0.010** | **6.8** | **0.00** | **0.86** | **0.37** | **0.00** |
| S_RI1_2013 | 2013 | INN S | **imazamox** | **0.022** | **6.8** | **0.00** | **0.98** | **0.37** | **0.00** |
| B_RI2_2016 | 2016 | INN W | **prochloraze** | **0.100** | **6.9** | **0.00** | **1.00** | **0.37** | **1.00** |
| M_RI1_2015 | 2015 | INN M | **mésotrione** | **0.140** | **6.9** | **0.24** | **0.39** | **0.37** | **0.80** |
| M_RI2_2017 | 2017 | INN M | **mesotrione** | **0.150** | **6.9** | **0.24** | **0.46** | **0.37** | **0.80** |
| M_RR_2015 | 2015 | REF M | **mesotrione** | **0.140** | **6.9** | **0.24** | **0.39** | **0.45** | **0.80** |
| B_RI3_2015 | 2015 | INN W | **iodosulfuron-méthyl-sodium** | **0.006** | **6.9** | **0.00** | **0.44** | **0.37** | **0.00** |
| M_RI2_2014 | 2014 | INN M | **mesotrione** | **0.120** | **6.9** | **0.24** | **0.38** | **0.37** | **0.80** |
| S_RI3_2014 | 2014 | INN S | **imazamox** | **0.013** | **6.9** | **0.00** | **0.94** | **0.37** | **0.00** |
| M_RR_2017 | 2017 | REF M | **mesotrione** | **0.111** | **7.0** | **0.24** | **0.47** | **0.45** | **0.80** |
| M_RI3_2016 | 2016 | INN M | **nicosulfuron** | **0.018** | **7.1** | **0.00** | **1.00** | **0.37** | **0.00** |
| M_RI1_2018 | 2018 | INN M | **mesotrione** | **0.099** | **7.1** | **0.24** | **0.53** | **0.37** | **0.80** |
| M_RR_2015 | 2015 | REF M | **mesotrione** | **0.097** | **7.1** | **0.24** | **0.58** | **0.45** | **0.80** |
| M_RR_2014 | 2014 | REF M | **mesotrione** | **0.113** | **7.1** | **0.24** | **0.68** | **0.45** | **0.80** |
| M_RI3_2016 | 2016 | INN M | **dimethenamid-p** | **0.281** | **7.1** | **0.60** | **0.58** | **0.37** | **0.80** |
| B_RI2_2016 | 2016 | INN W | **2,4 D** sel dimethylamine | **0.588** | **7.1** | **1.00** | **0.38** | **0.37** | **0.00** |
| S_RI1_2013 | 2013 | INN S | **imazamox** | **0.011** | **7.2** | **0.00** | **1.00** | **0.37** | **0.00** |
| B_RI1_2014 | 2014 | INN W | **prochloraze** | **0.080** | **7.3** | **0.00** | **1.00** | **0.37** | **1.00** |
| M_RI3_2016 | 2016 | INN M | **mésotrione** | **0.097** | **7.4** | **0.24** | **0.83** | **0.37** | **0.80** |
| M_RI3_2016 | 2016 | INN M | **2,4 D** sel dimethylamine | **0.300** | **7.5** | **1.00** | **0.26** | **0.37** | **0.00** |
| M_RI3_2013 | 2013 | INN M | **acétochlore** | **1.400** | **7.6** | **0.99** | **0.46** | **0.37** | **0.99** |
| M_RI3_2016 | 2016 | INN M | **nicosulfuron** | **0.008** | **7.7** | **0.00** | **1.00** | **0.37** | **0.00** |
| M_RI2_2017 | 2017 | INN M | **mésotrione** | **0.075** | **7.7** | **0.24** | **0.70** | **0.37** | **0.80** |
| M_RR_2018 | 2018 | REF M | **mésotrione** | **0.071** | **7.7** | **0.24** | **0.60** | **0.45** | **0.80** |
| M_RR_2015 | 2015 | REF M | **isoxaflutole** | **1.275** | **7.8** | **1.00** | **0.39** | **0.45** | **1.00** |
| B_RI2_2013 | 2013 | INN W | **tébuconazole** | **0.060** | **7.8** | **0.00** | **1.00** | **0.37** | **1.00** |
| M_RR_2013 | 2013 | REF M | **mésotrione** | **0.071** | **7.9** | **0.24** | **0.73** | **0.45** | **0.80** |
| B_RI2_2016 | 2016 | INN W | **cyproconazole** | **0.050** | **8.0** | **0.00** | **1.00** | **0.37** | **1.00** |
| M_RI1_2018 | 2018 | INN M | **glyphosate** | **0.360** | **8.0** | **0.96** | **0.82** | **0.37** | **0.00** |
| B_RI2_2016 | 2016 | INN W | **tébuconazole** | **0.050** | **8.0** | **0.00** | **1.00** | **0.37** | **1.00** |
| M_RR_2014 | 2014 | REF M | **bénoxacor** | **0.019** | **8.0** | **0.00** | **0.68** | **0.45** | **1.00** |
| M_RR_2013 | 2013 | REF M | **mésotrione** | **0.063** | **8.1** | **0.24** | **0.80** | **0.45** | **0.80** |
| M_RI3_2013 | 2013 | INN M | **mésotrione** | **0.063** | **8.1** | **0.24** | **0.83** | **0.37** | **0.80** |
| M_RI3_2013 | 2013 | INN M | **rimsulfuron** | **0.006** | **8.1** | **0.00** | **1.00** | **0.37** | **0.00** |
| M_RR_2015 | 2015 | REF M | **mésotrione** | **0.049** | **8.2** | **0.24** | **0.58** | **0.45** | **0.80** |
| B_RI1_2014 | 2014 | INN W | **tébuconazole** | **0.040** | **8.2** | **0.00** | **1.00** | **0.37** | **1.00** |
| M_RR_2013 | 2013 | REF M | **rimsulfuron** | **0.006** | **8.3** | **0.00** | **1.00** | **0.45** | **0.00** |
| B_RI1_2014 | 2014 | INN W | **phosphate ferrique** | **0.208** | **8.5** | **1.00** | **0.00** | **0.37** | **1.00** |
| M_RR_2018 | 2018 | REF M | **dicamba** | **0.135** | **8.5** | **1.00** | **0.60** | **0.37** | **0.00** |
| M_RR_2015 | 2015 | REF M | **dicamba** | **0.140** | **8.6** | **1.00** | **0.58** | **0.45** | **0.00** |
| M_RI2_2017 | 2017 | INN M | **dicamba** | **0.135** | **8.7** | **1.00** | **0.70** | **0.37** | **0.00** |
| M_RI1_2018 | 2018 | INN M | **dicamba** | **0.135** | **8.8** | **1.00** | **0.72** | **0.37** | **0.00** |
| M_RI3_2013 | 2013 | INN M | **fluroxypyr** | **0.024** | **8.8** | **0.31** | **1.00** | **0.37** | **0.00** |
| M_RR_2014 | 2014 | REF M | **mésotrione** | **0.036** | **8.8** | **0.24** | **0.86** | **0.45** | **0.80** |
| M_RR_2013 | 2013 | REF M | **fluroxypyr** | **0.024** | **8.9** | **0.31** | **1.00** | **0.45** | **0.00** |
| M_RR_2018 | 2018 | REF M | **dicamba** | **0.130** | **8.9** | **1.00** | **0.72** | **0.45** | **0.00** |
| S_RI1_2016 | 2016 | INN S | **pendiméthaline** | **0.600** | **8.9** | **1.00** | **0.65** | **0.37** | **1.00** |
| M_RI1_2015 | 2015 | INN M | **mésotrione** | **0.023** | **9.0** | **0.24** | **0.79** | **0.37** | **0.80** |
| M_RR_2013 | 2013 | REF M | **dicamba** | **0.173** | **9.0** | **1.00** | **0.80** | **0.45** | **0.00** |
| M_RR_2017 | 2017 | REF M | **dicamba** | **0.130** | **9.0** | **1.00** | **0.77** | **0.45** | **0.00** |
| M_RI3_2016 | 2016 | INN M | **dicamba** | **0.140** | **9.0** | **1.00** | **0.83** | **0.37** | **0.00** |
| M_RI3_2013 | 2013 | INN M | **dicamba** | **0.130** | **9.0** | **1.00** | **0.83** | **0.37** | **0.00** |
| M_RI2_2014 | 2014 | INN M | **mésotrione** | **0.024** | **9.1** | **0.24** | **0.86** | **0.37** | **0.80** |
| S_RI1_2016 | 2016 | INN S | **clomazone** | **0.054** | **9.2** | **0.84** | **0.65** | **0.37** | **0.87** |
| M_RI3_2016 | 2016 | INN M | **mésotrione** | **0.045** | **9.3** | **0.24** | **1.00** | **0.37** | **0.80** |
| M_RR_2016 | 2016 | REF M | **dicamba** | **0.093** | **9.4** | **1.00** | **0.83** | **0.45** | **0.00** |
| M_RI2_2014 | 2014 | INN M | **aclonifen** | **0.250** | **9.4** | **1.00** | **0.38** | **0.37** | **1.00** |
| M_RI3_2016 | 2016 | INN M | **pendiméthaline** | **0.330** | **9.4** | **1.00** | **0.58** | **0.37** | **1.00** |
| M_RI3_2013 | 2013 | INN M | **aclonifen** | **0.200** | **9.6** | **1.00** | **0.46** | **0.37** | **1.00** |
| M_RI3_2016 | 2016 | INN M | **mésotrione** | **0.020** | **9.8** | **0.24** | **1.00** | **0.37** | **0.80** |
| S_RI2_2018 | 2018 | INN S | **pendiméthaline** | **0.114** | **9.9** | **1.00** | **0.60** | **0.37** | **1.00** |
| M_RI3_2013 | 2013 | INN M | **isoxaflutole** | **0.030** | **10.0** | **1.00** | **0.46** | **0.37** | **1.00** |
| M_RI3_2013 | 2013 | INN M | **cyperméthrine** | **0.000** | **10.0** | **1.00** | **0.92** | **0.37** | **1.00** |
| M_RI3_2013 | 2013 | INN M | **phosphate ferrique** | **0.195** | **10.0** | **1.00** | **1.00** | **0.37** | **1.00** |
| M_RI3_2013 | 2013 | INN M | **dicamba** | **0.038** | **10.0** | **1.00** | **1.00** | **0.37** | **0.00** |
| M_RI3_2013 | 2013 | INN M | **florasulam** | **0.000** | **10.0** | **1.00** | **1.00** | **0.37** | **0.00** |
| M_RI2_2014 | 2014 | INN M | **isoxaflutole** | **0.038** | **10.0** | **1.00** | **0.38** | **0.37** | **1.00** |
| M_RI2_2014 | 2014 | INN M | **téfluthrine** | **0.000** | **10.0** | **1.00** | **0.75** | **0.37** | **1.00** |
| M_RI1_2015 | 2015 | INN M | **cyperméthrine** | **0.000** | **10.0** | **1.00** | **0.78** | **0.37** | **1.00** |
| M_RI1_2015 | 2015 | INN M | **isoxaflutole** | **0.075** | **10.0** | **1.00** | **0.39** | **0.37** | **1.00** |
| M_RI1_2015 | 2015 | INN M | **phosphate ferrique** | **0.173** | **10.0** | **1.00** | **1.00** | **0.37** | **1.00** |
| M_RI1_2015 | 2015 | INN M | **métaldéhyde** | **0.155** | **10.0** | **1.00** | **1.00** | **0.37** | **1.00** |
| M_RI1_2015 | 2015 | INN M | **phosphate ferrique** | **0.045** | **10.0** | **1.00** | **1.00** | **0.37** | **1.00** |
| M_RI3_2016 | 2016 | INN M | **métaldéhyde** | **0.200** | **10.0** | **1.00** | **1.00** | **0.37** | **1.00** |
| M_RI3_2016 | 2016 | INN M | **dicamba** | **0.086** | **10.0** | **1.00** | **1.00** | **0.37** | **0.00** |
| M_RI3_2016 | 2016 | INN M | **dicamba** | **0.072** | **10.0** | **1.00** | **1.00** | **0.37** | **0.00** |
| M_RI2_2017 | 2017 | INN M | **cyperméthrine** | **0.000** | **10.0** | **1.00** | **0.92** | **0.37** | **1.00** |
| S_RI3_2014 | 2014 | INN S | **phosphate ferrique** | **0.119** | **10.0** | **1.00** | **1.00** | **0.37** | **1.00** |
| S_RI3_2014 | 2014 | INN S | **propaquizafop** | **0.042** | **10.0** | **1.00** | **0.94** | **0.37** | **1.00** |
| S_RI3_2017 | 2017 | INN S | **quizalofop ethyl** -d | **0.048** | **10.0** | **1.00** | **0.79** | **0.37** | **1.00** |
| S_RI2_2018 | 2018 | INN S | **fluazifop-p-butyl** | **0.060** | **10.0** | **1.00** | **0.96** | **0.37** | **1.00** |
| B_RI3_2015 | 2015 | INN W | **fénoxaprop-p-éthyl** | **0.048** | **10.0** | **1.00** | **0.44** | **0.37** | **1.00** |
| B_RI3_2015 | 2015 | INN W | **méfenpyr-diéthyl** | **0.018** | **10.0** | **1.00** | **0.44** | **0.37** | **1.00** |
| B_RI2_2016 | 2016 | INN W | **pinoxaden** | **0.028** | **10.0** | **1.00** | **0.38** | **0.37** | **1.00** |
| B_RI2_2016 | 2016 | INN W | **cloquintocet-mexyl** | **0.007** | **10.0** | **1.00** | **0.38** | **0.37** | **1.00** |
| B_RI2_2016 | 2016 | INN W | **chlorothalonil** | **0.375** | **10.0** | **1.00** | **1.00** | **0.37** | **1.00** |
| B_RI2_2016 | 2016 | INN W | **propiconazole** | **0.063** | **10.0** | **1.00** | **1.00** | **0.37** | **1.00** |
| M_RR_2013 | 2013 | REF M | **cyperméthrine** | **0.000** | **10.0** | **1.00** | **0.92** | **0.45** | **1.00** |
| M_RR_2013 | 2013 | REF M | **dicamba** | **0.038** | **10.0** | **1.00** | **1.00** | **0.45** | **0.00** |
| M_RR_2013 | 2013 | REF M | **florasulam** | **0.000** | **10.0** | **1.00** | **1.00** | **0.45** | **0.00** |
| M_RR_2014 | 2014 | REF M | **téfluthrine** | **0.000** | **10.0** | **1.00** | **0.75** | **0.45** | **1.00** |
| M_RR_2016 | 2016 | REF M | **dicamba** | **0.043** | **10.0** | **1.00** | **1.00** | **0.45** | **0.00** |

**References**

Agreste (2020) Cultures développées (hors fourrage, prairies, fruits, fleurs et vigne). https://agreste.agriculture.gouv.fr/agreste-saiku/?plugin=true&query=query/open/SAANR_DEVELOPPE_2#query/open/SAANR_DEVELOPPE_2

Alletto L, Coquet Y, Benoit P, et al (2010) Tillage management effects on pesticide fate in soils. A review. Agron Sustain Dev 30:367–400. https://doi.org/10.1051/agro/2009018

Arcand MM, Knight JD, Farrell RE, 2013. Estimating belowground nitrogen inputs of pea and canola and their contribution to soil inorganic N pools using 15N labeling. Plant Soil 371: 67–80

Arvalis-Institut du Végétal I, CTIFL, IFIP, et al (2020) Guide GES’TIM+ Juin 2020. Projet réalisé par Arvalis, en partenariat avec l’Idèle, le Ctifl, l’Ifv, l’Itavi, l’Ifip et Terres Inovia. Avec la participation financière de l’ADEME - Agence de la transition écologique. GES’TIM+ : la référence méthodologique. https://www.arvalisinstitutduvegetal.fr/file/galleryelement/pj/65/09/79/f3/gestim7645230857668885746.pdf. Accessed 31 Aug 2021

Bell, C.W., Asao, S., Calderon, F., Wolk, B., Wallenstein, M.D., 2015. Plant nitrogen uptake drives rhizosphere bacterial community assembly during plant growth. Soil Biol. Biochem. 85, 170–182. https://doi.org/10.1016/J.SOILBIO.2015.03.006

Bockstaller C, Galland V, Avadí A (2022) Modelling direct field nitrogen emissions using a semi-mechanistic leaching model newly implemented in Indigo-N v3. Ecol Modell 472:110109. https://doi.org/10.1016/J.ECOLMODEL.2022.110109

Bockstaller C, Girardin P, Van der Werf HGM (1997) Use of agro-ecological indicators for the evaluation of farming systems. Eur J Agron 7:261–270

Bockstaller C, Guichard L, Keichinger O, et al (2009) Comparison of methods to assess the sustainability of agricultural systems. A review. Agron Sustain Dev 29:223–235. https://doi.org/doi:10.1051/agro:2008058

Bockstaller C, Guichard L, Makowski D, et al (2008) Agri-environmental indicators to assess cropping and farming systems. A review. Agron Sustain Dev 28:139–149. https://doi.org/doi:10.1051/agro:2007052

Bockstaller C, Lassere-Joulin F, Slezack-Deschaumes S, et al (2011) Assessing biodiversity in arable farmland by means of indicators: an overview. Oléagineux Corps gras Lipides 18:137–144. https://doi.org/10.1684/ocl.2011.0381

Bohanec M, Messean A, Scatasta S, et al (2008) A qualitative multi-attribute model for economic and ecological assessment of genetically modified crops. Ecol Modell 215:247–261. https://doi.org/doi:10.1016/j.ecolmodel.2008.02.016

Bouwman AF (1996) Direct emission of nitrous oxide from agricultural soils. Nutr Cycl Agroecosystems 46:53–70

Brookes, P.C., Landman, A., Pruden, G., Jenkinson, D.S., 1985. Chloroform fumigation and the release of soil nitrogen: A rapid direct extraction method to measure microbial biomass nitrogen in soil. Soil Biol. Biochem. 17, 837–842. https://doi.org/10.1016/0038-0717(85)90144-0

Bureau de Coordination du Machinisme Agricole (BCMA) (2012) Simcoguide online decision tool. http://simcoguide.pardessuslahaie.net/#accueil. Accessed 12 Apr 2013

Craheix D (2015) CRITER: Un outil de caractérisation des performances de systèmes de culture. http://wiki.inra.fr/wiki/deximasc/package+MASC/CRITER. Accessed 29 Mar 2021

Craheix D, Angevin F, Bergez JE, et al (2011) MASC 2.0, Un outil pour l’analyse de la contribution des systèmes de culture au développement durable. Jeu complet de fiches critères de MASC 2.0

Craheix D, Angevin F, Doré T, de Tourdonnet S (2016) Using a multicriteria assessment model to evaluate the sustainability of conservation agriculture at the cropping system level in France. Eur J Agron 76:75–86. https://doi.org/10.1016/J.EJA.2016.02.002

Dairon R, Dutertre A, Tournebize J, et al (2017) Long-term impact of reduced tillage on water and pesticide flow in a drained context. Environ Sci Pollut Res 24:6866–6877. https://doi.org/10.1007/s11356-016-8123-x

Davis AS, Hill JD, Chase CA, et al (2012) Increasing Cropping System Diversity Balances Productivity, Profitability and Environmental Health. PLoS One 7:e47149. https://doi.org/10.1371/journal.pone.0047149

Deytieux V, Munier-Jolain N, Caneill J (2016) Assessing the sustainability of cropping systems in single- and multi-site studies. A review of methods. Eur J Agron 72:107–126. https://doi.org/10.1016/j.eja.2015.10.005

Durand P, Gascuel-Odoux C, Cordier MO (2002) Parametrisation of hydrological models: a review and lesson learned from studies on an agricultural catchment (Naizin, France). Agronomie 22:217–228

Elias D, Wang L, Jacinthe PA (2018) A meta-analysis of pesticide loss in runoff under conventional tillage and no-till management. Environ Monit Assess 190:1–17. https://doi.org/10.1007/s10661-017-6441-1

Germon JC, Hénault C, Garrido F, Reau R (1999) Mécanisme de production, régulation et possibilités de limitation des émissions de N2O à l’échelle agronomique. Comptes Rendus l’Académie l’Agriculture Fr 85:148–162

Giuliano S, Ryan MR, Véricel G, et al (2016) Low-input cropping systems to reduce input dependency and environmental impacts in maize production: A multi-criteria assessment. Eur J Agron 76:160–175. https://doi.org/10.1016/J.EJA.2015.12.016

Gravesen, L., 2003. The Treatment Frequency Index: an indicator for pesticide use and dependancy as well as overall load on the environment. In: Pesticide Action Network Europe, Pure Conference, Copenhagen Danemark.

Joergensen, R.G., 1996. Quantification of the microbial biomass by determining ninhydrin-reactive N. Soil Biol. Biochem. 28, 301–306. https://doi.org/10.1016/0038-0717(95)00141-7

Ladd, J.N., Butler, J.H.A., 1972. Short-term assays of soil proteolytic enzyme activities using proteins and dipeptide derivatives as substrates. Soil Biol. Biochem. 4, 19–30. https://doi.org/10.1016/0038-0717(72)90038-7

Lechenet M, Bretagnolle V, Bockstaller C, et al (2014) Reconciling pesticide reduction with economic and environmental sustainability in arable farming. PLoS One 9:1–10

Lindahl AML, Bockstaller C (2012) An indicator of pesticide leaching risk to groundwater. Ecol Indic 23:95–108. https://doi.org/doi:10.1016/j.ecolind.2012.03.014

Massot P, Deytieux V, Fonteny C, et al (2016) Des scénarios de prix pour évaluer les performances économiques des systèmes de culture. In: Poster, Systèmes de culture innovants : concevoir, former, accompagner, 17th May 2016, Paris. Paris , France

Melland AR, Silburn DM, McHugh AD, et al (2015) Spot Spraying Reduces Herbicide Concentrations in Runoff. J Agric Food Chem 64:4009–4020. https://doi.org/10.1021/ACS.JAFC.5B03688

Ministère de l’Agriculture et de l’Alimentation (2019) Émissions de gaz à effet de serre : l’agriculture française respecte les objectifs de la Stratégie nationale bas-carbone. https://agriculture.gouv.fr/emissions-de-gaz-effet-de-serre-lagriculture-francaise-respecte-les-objectifs-de-la-strategie. Accessed 27 Jun 2021

Oenema O, Kros H, de Vries W (2003) Approaches and uncertainties in nutrient budgets: implications for nutrient management and environmental policies. Eur J Agron 20:3–16

Pervanchon F, Bockstaller C, Girardin P (2002) Assessment of energy use in arable farming systems by means of an agro-ecological indicator: the energy indicator. Agric Syst 72:149–172

Pierlot F, Marks-Perreau J, Réal B, et al (2017) Predictive quality of 26 pesticide risk indicators and one flow model: A multisite assessment for water contamination. Sci Total Environ 605:655–665. https://doi.org/10.1016/j.scitotenv.2017.06.112

Pierlot F, Marks-Perreau J, Soulé E, et al (2023) An indicator to assess risks on water and air of pesticide spraying in crop fields. Sci Total Environ (in press):

R Core Team (2013) R: A Language and Environment for Statistical Computing. R Foundation forStatistical Computing, Vienna. http://www.R-project.org/

Roger-Estrade J, Richard G, Caneill J, et al (2004) Morphological characterisation of soil structure in tilled fields: From a diagnosis method to the modelling of structural changes over time. Soil Tillage Res 79:33–49. https://doi.org/10.1016/j.still.2004.03.009

Romillac N, Piutti S, Amiaud B, Slezack-Deschaumes S (2015) Influence of pea root traits modulating soil bioavailable C and N effects upon ammonification activity. Soil Biol Biochem 90:148–151. https://doi.org/10.1016/J.SOILBIO.2015.07.019

Romillac, N., Slezack-Deschaumes, S., Amiaud, B., Piutti, S., 2023. Soil Microbial Communities Involved in Proteolysis and Sulfate-Ester Hydrolysis Are More Influenced by Interannual Variability than by Crop Sequence. Agron. 2023, Vol. 13, Page 180 13, 180. https://doi.org/10.3390/AGRONOMY13010180

Soman C, Li D, Wander MM, Kent AD (2017) Long-term fertilizer and crop-rotation treatments differentially affect soil bacterial community structure. Plant Soil 413: 145–159

Sinsabaugh RL, Lauber CL, Weintraub MN et al (2008). Stoichiometry of soil enzyme activity at global scale. Ecology Letters 11:1252–1264

Snapp SS, Gentry LE, Harwood R (2010) Management intensity - not biodiversity - the driver of ecosystem services in a long-term row crop experiment. Agric Ecosyst Environ 138:242–248. https://doi.org/10.1016/j.agee.2010.05.005

Taureau JC, Gitton C, Laurent F, et al (1996) Calcul de la fertilisation azotée des cultures annuelles. COMIFER, Paris

ten Berge HFM (2002) A review of potential indicators for nitrate loss from cropping and farming systems in the Netherlands. 168

Uzoh IM, Igwe CA, Okebalama CB, Babalola OO (2019) Legume-maize rotation effect on maize productivity and soil fertility parameters under selected agronomic practices in a sandy loam soil. Scientific Reports 9: 8539.

Vance ED, Brookes PC, Jenkinson DS (1987) Microbial biomass measurements in forest soils: The use of the chloroform fumigation-incubation method in strongly acid soils. Soil Biol Biochem 19:697–702. https://doi.org/10.1016/0038-0717(87)90051-4

van der Werf HMG, Zimmer C (1998) An indicator of pesticide environmental impact based on a fuzzy expert system. Chemosphere 36:2225–2249

van Kessel C, Venterea R, Six J, et al (2013) Climate, duration, and N placement determine N _2_ O emissions in reduced tillage systems: a meta-analysis. Glob Chang Biol 19:33–44. https://doi.org/10.1111/j.1365-2486.2012.02779.x

Vasileiadis VP, Dachbrodt-Saaydeh S, Kudsk P, et al (2017) Sustainability of European winter wheat- and maize-based cropping systems: Economic, environmental and social ex-post assessment of conventional and IPM-based systems. Crop Prot 97:60–69. https://doi.org/10.1016/J.CROPRO.2016.11.002

Woodrow JSE, Sieber JSN, Baker L (1997) Correlation techniques for estimating pesticide volatilization flux and downwind concentrations. Environ Sci Technol 31:523–529
